# Social Factors Influencing Russian Male Alcohol Use over the Life Course: A Qualitative Study Investigating Age Based Social Norms, Masculinity, and Workplace Context

**DOI:** 10.1371/journal.pone.0142993

**Published:** 2015-11-17

**Authors:** Katherine Keenan, Lyudmila Saburova, Natalia Bobrova, Diana Elbourne, Sarah Ashwin, David A. Leon

**Affiliations:** 1 London School of Economics, Houghton Street, London, United Kingdom; 2 Izhevsk State Technical University, 7 Studencheskaya Street, Izhevsk, Russia; 3 London School of Hygiene and Tropical Medicine, Keppel Street, London, United Kingdom; Harvard Medical School, UNITED STATES

## Abstract

The massive fluctuations occurring in Russian alcohol-related mortality since the mid-1980s cannot be seen outside of the context of great social and economic change. There is a dearth of qualitative studies about Russian male drinking and especially needed are those that address social processes and individual changes in drinking. Conducted as part of a longitudinal study on men’s alcohol consumption in Izhevsk, this qualitative study uses 25 semi-structured biographical interviews with men aged 33–60 years to explore life course variation in drinking. The dominant pattern was decreasing binge and frequent drinking as men reached middle age which was precipitated by family building, reductions in drinking with work colleagues, and health concerns. A minority of men described chaotic drinking histories with periods of abstinence and heavy drinking. The results highlight the importance of the blue-collar work environment for conditioning male heavy drinking in young adulthood through a variety of social, normative and structural mechanisms. Post-Soviet changes had a structural influence on the propensity for workplace drinking but the important social function of male drinking sessions remained. Bonding with workmates through heavy drinking was seen as an unavoidable and essential part of young men’s social life. With age peer pressure to drink decreased and the need to perform the role of responsible breadwinner put different behavioural demands on men. For some resisting social pressure to drink became an important site of self-determination and a mark of masculine maturity. Over the lifetime the place where masculine identity was asserted shifted from the workplace to the home, which commonly resulted in a reduction in drinking. We contribute to existing theories of Russian male drinking by showing that the performance of age-related social roles influences Russian men’s drinking patterns, drinking contexts and their attitudes. Further research should be conducted investigating drinking trajectories in Russian men.

## Introduction

Heavy and hazardous alcohol use is an important driver of the Russian male mortality crisis, and since 1980 there have been large parallel fluctuations in population-level drinking and premature death [1–4]. Previous studies have addressed the macro-level factors affecting population-level drinking [5,6] but very few have addressed the social and contextual factors that are associated with individual change in drinking over the life course [7,8]. Qualitative research methods are particularly well suited for understanding these social processes, but there is a dearth of these focussing on Russian men’s drinking, with some exceptions [8,9]. This study addresses this research gap through a qualitative investigation of the life-course dynamics of Russian men’s alcohol use.

Previous qualitative studies have highlighted how Russian male heavy drinking is related to demonstrations of masculinity, and is more common in male-dominated environments such as blue-collar workplaces [8,10,11]. This is consistent with findings from quantitative surveys that hazardous drinking in Russia is more common among men with lower socio-economic status [12–16]. However, relatively little is known about how gender, social position, and context combine to produce high rates of hazardous drinking. We have previously used qualitative biographical interviews [17] to explore drinking patterns of men who had died of alcohol-related causes, which highlighted the interaction between norms, biosocial factors and alcohol use [8]. In this study we use similar methods to explore the longitudinal dynamics of Russian male drinking patterns over the previous 10–20 year period. We interviewed working-age men in Izhevsk to investigate the perceived events that have influenced their drinking pattern, paying particular attention to accounts of social processes, social context and norms. We discuss the results in the context of post-Soviet social upheaval while drawing on sociological theories of masculinity, ageing and social reproduction.

## Background

### The social context of Russian alcohol consumption

Any account of recent Russian drinking is impossible without considering the social context of the USSR and its subsequent collapse in 1991. Russian men born between 1950–70 (the cohorts in this study) would have grown up in relative stability and had their schooling, employment, healthcare, and housing provided, although frequent supply shortages led to reliance on social exchange networks known as *blat* [18]. However during the collapse of the USSR in 1991 and the unstable post-Soviet period of the 1990s, Russians experienced a series of deep social, political and economic changes characterised by increasing income inequality, increasing criminality [19,20], and for most people, a chaotic struggle to survive [21]. In the 1990s Russia suffered a severe economic crisis where levels of poverty and unemployment soared [22], many suffered wage delays, and many lost their savings. Rapid changes in the nature of employment, industry and the meaning of networks meant that older generations had difficulty adjusting, and ended up feeling displaced and ‘unneeded’ [21]. Since the 2000s the economy has prospered due to oil and gas exports; correspondingly employment rates and the standard of living have improved making life more stable for Russian citizens [23].

### Patterns of alcohol consumption in Russia

For centuries Russia has been noted for its heavy drinking [24] and by the start of the 1900s, drinking was commonly regarded as ‘part of the national character of the Russian people’ [25]. The Soviet government did little to curb rising consumption, in part because alcohol excise duty contributed significantly to the government budget [26]. From the mid-1980s to today rates of harmful alcohol consumption have fluctuated dramatically. Gorbachev’s anti-alcohol campaign from 1985–87 [27] successfully reduced consumption but immediately afterwards in the early 1990s there was a dramatic increase in alcohol-related mortality, fuelled by the sudden availability of cheap alcohol that followed market deregulation. A further spike occurred in 1998 related to the social effects of the Rouble crisis [28]. Since 2005 alcohol-related mortality has declined partly due to progressive restrictions on alcohol production and its availability [29]. However, in Russia and the former Soviet Union alcohol use still accounts for 10–14% of total mortality [30], one in every five male deaths, and a quarter of total disease burden, more than any other world region [31].

Per capita annual consumption in Russia is high, estimated at 15.7 litres of ethanol in 2011 [32], and the drinking pattern is characterised by a high proportion of alcohol drunk as spirits [33], a norm of drinking to intoxication and binge drinking [8,24], and the relatively common use of non-beverage alcohols (also known as surrogates) like eau de cologne and medicinal tinctures [14,34]. Men drinking more frequently, more as spirits and are several times more likely to binge drink than women [13,16,35,36]. In a cross-sectional survey from Izhevsk in the 2000s approximately 10% of men reported going on *‘zapoi’* (defined as period of two or more days of continuous drunkenness where a person is withdrawn from social life) and 7% drinking non-beverage alcohols in the last year [14]. Treatment for alcohol problems relies heavily on suggestion-based methods such as ‘coding’ (*kodirovanie*) usually provided privately by narcologists (Russian addictions specialists) [37].

### Masculinity and drinking in Russia

Several studies have explored gender ideology and heavy drinking in Russia [35,38–40]. Men’s heavy drinking is commonly perceived as ‘natural’, something that ‘real men’ do, in keeping with their breadwinner role, and there is a strong element of machismo to Russian male binge drinking [8,9]. Some argue that Russian male heavy drinking reinforces aspects of ‘hegemonic masculinity’ particularly in working class work environments [38]. Typically Russian women (particularly wives) have a duty to monitor and control men’s drinking, a form of gendered caring which constructs femininity in opposition to masculinity [41,42].

In Russia, alcohol is perceived to be the primary male stress reliever [15,39,43]; by contrast women have an ability to cope without resorting to drink [44]. This might explain why disruptions in a man’s work life were seen as understandable cause of heavy drinking [8], and partly why rates of hazardous drinking are highest amongst those suffering social stress such as unemployment and divorce [45]. Male hazardous drinking increased in the early 1990s, coinciding with high rates of unemployment, wage delays and changes to working patterns [45].

In Russia alcohol also plays an important role in work, business and exchange [8,46,47]. Research from the 1990s shows that the workplace was a more important drinking context in Russia than in other countries [48], with 20% of Russians reporting drinking at work, compared to only 2% of Finns [48]. Ethnographic studies showed how vodka drinking in all-male work brigades (known in the Soviet period as ‘*kollektivs*’) strengthened bonds among workers [11,49]. A study from the 2000s found that *zapois* often started with work colleagues, and that particular used alcohol instead of payment [8]. Despite changes to employment in the post-Soviet period, studies suggest that workplace drinking and the perception that it promotes social solidarity continue in many industrial sectors [10,11]. Focussing on the blue-collar workplace is particularly important given the concentration of hazardous drinking in those with lower socio-economic position [14].

### Theoretical framework for Russian male drinking

Previous studies theorising Russian men’s heavy drinking have employed the concepts of ‘hegemonic masculinity’[38] and ‘health lifestyle theory’ [50]. The former argued that historically ritualised heavy drinking allowed working class men to access dominant masculine ideals [38]. Cockerham and colleagues have applied ‘health lifestyle theory’[50] which argues that Russian mens’ health behaviours (including their drinking) are primarily determined by the ‘habitus’[51], allowing little individual agency [50]. Bourdieu defined habitus as “systems of durable, transposable dispositions”, which both structure social life and interaction, and are constantly being reproduced by social activities. This passive portrayal of Russian men echoes the notion of ‘homo soveticus’- a person whose mentality was a legacy of the Soviet ideals of state paternalism and is devoid of individuality. Although both theories are supported empirically [39,43], both emphasise cultural reproduction and do not include convincing accounts of how change occurs, which is important for this study. To overcome this limitation one approach is to focus on aspects of identity that vary over the life course such as age. In a recent study in Scotland respondents described their drinking as passing through developmental stages [52] each with expectations of age-appropriate behaviour. In Russia the concept of ‘doing age’ has been used to theorise grandmothers’ care-giving [53] and this has been extended to ‘doing *gendered* age’ in a recent Russian study on patterns of gendered reciprocity [54]. These contributions emphasise the intersection of gender and age norms, such that the gendered expectations to which men and women are expected to conform modify as they age. The performance of age- and gender-appropriate behaviour could feasibly be applied to Russian men’s drinking, allowing us to reconcile theories of masculinity and habitus with change over the life course.

## Aims of This Study

This qualitative study aimed to explore self-conceptualisations of changes in Russian men’s drinking patterns over their life time. Specifically, the objectives are:

- To identify the circumstances of change in men’s drinking careers, and perceptions about the reasons for such changes;
- To explore how the blue-collar workplace and other social contexts, and the norms surrounding them are understood to affect drinking patterns and behaviours.

## Materials and Methods

The qualitative study was carried out between 2009 and 2012 in Izhevsk, the capital of the Udmurt Republic, on the western side of the Ural Mountains, approximately 1000 km east of Moscow. It is a medium sized industrial city, with a population of about 630,000 (2010 census). Soviet employment in Izhevsk had been dominated by a few heavy industrial plants. During the 1990s plants were rapidly privatised and there was a 10-fold growth in private companies [55], and unemployment trebled. Today a large proportion of the population are still employed in heavy industry (in 1993 this was 33% of the workforce; in 2011 it had declined only slightly to 30%) [55,56].

These in-depth interviews were collected as part of the Izhevsk Family Study (IFS), which is described in detail elsewhere [1], and is a cohort study of working-age men. The men in our study were interviewed first in 2003–05 (IFS-1) and for the second time (IFS-2) between 2007 and 2009. In the second interview some participants were randomly allocated to participate in a motivational interviewing (MI) trial [57] and those who consented received up to four one-to-one sessions discussing their alcohol use (7 of the 25 men interviewed). The information in the main study was largely collected through a fully-structured questionnaire with questions about the man’s alcohol use, and his social, behavioural and health characteristics.

### Sample selection

The sampling frame consisted of 1,447 men who were interviewed twice before and had agreed to participate in future research. Purposive sampling was used to try to ensure we interviewed men with a range of drinking patterns and included increases, decreases and stability in alcohol consumption. To do this we used a question from the 2008–09 interview which asked “*Have there been any changes in your drinking behaviour since the last interview*?, with possible answers *“I used to be an abstainer*, *but now I drink alcohol/I used to drink alcohol*, *now I’m an abstainer/I used to drink alcohol*, *now I drink surrogates as well/I used to drink surrogates*, *now I drink alcoholic drinks but no surrogates/no change/other* (free text response)*”*. Initially, 37 respondents were randomly selected from across the categories above. 19 were successfully contacted by telephone or face-to-face and agreed to participate (the other 12 did not answer the telephone, or refused due to health problems or not having the time) and were interviewed between October 2009 and October 2010. After initial data coding, we identified the need to have more accounts of increases in drinking, and further randomly selected another six respondents from categories indicating such changes, all of whom agreed to participate. In total there are 25 interviews conducted between October 2009 and February 2012. We did not discuss the transcripts and research findings with the participants after the interview itself.

### Interview methods

The study was approved by the ethical committees of the London School of Hygiene and Tropical Medicine and the Izhevsk Medical Academy. Verbal consent was obtained before each interview commenced and participants were assured that their data would be treated confidentially. This was consistent with the exclusive use of verbal consent in the Izhevsk studies [8] and was approved by the ethical committees on the basis that in Russia there remains a substantial reluctance to sign any official document for fear of the individual being held to account by the authorities for what they had said.

The interviews were conducted by five interviewers (2 male, 3 female) experienced in sociological interviewing. Four of the interviewers held qualifications in sociology (One had a PhD, the others had MSc or equivalent), and were employed by Izhevsk State University as researchers. All had received training and had experience at qualitative interviewing. The other interviewer was a psychiatrist (holding a medical degree) with expertise in qualitative interviewing and addiction treatment. All interviews were audio recorded and most took place one-one-one in the respondent’s home. Prior to interview the interviewers were given details of the interviewee’s name and address; all other information was elicited during interview. Our interviewing methodology was consistent with the biographical-narrative approach [58] which assumes that psychological and social phenomena are best understood as situated within a broader context of life history and they are partly constructed through narration. Therefore in order to understand alcohol use and life events we would need to enquire about the respondent’s entire life, and their perceptions of events and actions. The interviewers initially asked the respondents to describe events in their lives over the previous 8–10 years particularly calling attention to the period between the IFS first and second interviews. The interviewers also asked about the man’s health, which led onto asking about any significant changes in drinking over the men’s entire life course, meaning the narratives extended further back to include the Soviet period. The interviewers used a short pre-agreed topic guide to probe the respondent on changes in alcohol consumption that might be related to their family circumstances, their employment and workplace, and their health (Table 1). The interviews lasted anywhere between 15 minutes to one hour. Afterwards the audio recordings were transcribed verbatim.

**Table 1:** Topic guide used by interviewers in the study.

Theme	Subthemes
Important events or changes in previous 8–10 years	Family/household, work, health, economic problems, deaths or illnesses of family members
Work /financial situation	Employment history, how was affected by economic crises, retirement (pension), spouse or other family members’ employment
Friendship	Friends and work colleagues, frequency of meeting; changes in friendship group over life-course
Family	Where born/when moved to Izhevsk, who respondent lives with, spouse, children/grandchildren, relationship quality
Health	Serious health events/chronic illnesses, hospital stays, results of recent health checks, what things influence health
Health behaviours	Smoking, drinking and physical exercise history, diet; attitude to changes in drinking and smoking
Alcohol	Age started drinking; time(s) in life when drank heavily; types of beverages they prefer/preferred;surrogate use; *zapois*/heavy drinking episodes and reasons; who they drink with/drank with in the past; if any changes over life-course what they think its related to; whether they think their health is affected by alcohol use; periods of abstinence; dependency; treatment, how workplace/family/friends/other factors affect alcohol use; attitude to alcohol in their workplace; family members’ drinking and attitudes to drinking

### Coding and analysis

The coding was done independently by three different researchers (KK, LS and NB) using the original Russian transcripts in NVivo version 8 [59]. After each stage of coding the interpretations were discussed and any differences negotiated. Initially, the transcripts were categorised according to whether they gave accounts of increases, decreases, or stability in alcohol use. They were then open coded to identify cross-cutting reasons for changes which were arose from the data, such as health problems, peer pressure or family responsibilities. After that a deeper level of coding identified themes and motivations, and common conceptualisations of the role of alcohol in the life course. Any excerpts used for illustration were professionally translated into English (and checked for meaning with the original interviewer), and the interviewee number shown after each quotation. Where possible we also compared the narrated accounts of drinking and life events with those reported in the IFS-1 and IFS-2 study interviews.

## Results

### Sample characteristics

Table 2 shows the respondents’ age and socio-demographic factors taken from the self-reported questionnaire data collected at IFS-2 (2007–09) and the qualitative interview. At the time of qualitative interview, the men were aged between 32 to 60 years (median 56 years). All of the men were ethnic Russians. Three quarters of the men (19) had achieved secondary or ‘specialised’ secondary/professional education (as their highest level), and a minority had some higher education (6). At the qualitative interview 20 men (80%) were in regular paid work, and the rest unemployed, in irregular work or retired. Over half of the interviewees (14 men) were employed in industry, most as semi-skilled manual workers (machine operators, mechanics etc), but three were team supervisors. Of the remaining men, three were directors or business owners, and a further eight had unskilled professions such as drivers or security guards. The majority of the men (22) were living with their spouse or partner and only one of the men was childless. Self-reports of drinking at IFS-2 (for the previous year) showed that two of the men had abstained, four had drunk non-beverage alcohols in the previous year, and the remaining 19 men were non-hazardous beverage drinkers.

**Table 2:** Socio-demographic characteristics and drinking patterns of the 25 men interviewed in the study.

Characteristic	N	%
**Age** ^1^		
<55 years	9	36.0%
55–59 years	16	64.0%
**Highest educational level** ^2^		
Secondary	9	36.0%
Professional/specialised secondary school	10	40.0%
Higher	6	24.0%
**Employment status** ^1^		
Regular paid employment	20	80.0%
Unemployed/retired/other	5	20.0%
**Partnership status** ^1^		
Married/cohabiting	22	88.0%
Divorced/widowed/other	3	12.0%
**Reported drinking pattern 12 months before IFS-2 interview** ^2^ ^3^		
Abstainer	2	8.0%
Beverage alcohols only, non-hazardous drinker	19	76.0%
Non-beverage alcohol drinker/hazardous drinker	4	16.0%

^1^Self-reported at the qualitative interview.

^2^ Self-reported at IFS-2 (2007–09)

^3^ Hazardous drinking was indicated by reports of *zapoi*, or one of the following twice a week or more: falling asleep with clothes on because of being drunk, ‘excessive drunkenness’, or hangover.

Of the 25 interviews, 18 described decreases, two described recent increases and five described fluctuations between abstinence and drinking. We compared the qualitative descriptions of recent alcohol use with the reports of change, and the cross-sectional reports of alcohol use from the previous two interviews in 2004–05 and 2007–09, and found the patterns to be generally consistent. Any differences were among men who reported fluctuations in the qualitative interview, and it was possible that sudden changes were not well captured by the questionnaire.

### Decreases over time

The explanations for decreases in consumption could be grouped into three interrelated themes: work, family, and the process of ageing or maturing. Within these, there were external influences that were felt to be both controlling and empowering, through changing norms or social control, and internal motivations where the man felt he had made a conscious, personal decision. The dominant trajectory was heavy drinking in young adulthood or middle age, often associated with particular workplaces, or sets of work colleagues. With age this was replaced by increased emphasis on home and family which coincided with reductions and greater control over drinking.

#### Workplace influences

Periods of heavy drinking in young adulthood were often associated with particular workplaces or institutions (for example, the army), type of work or shift pattern (aircraft engineer, rotation in remote areas). Some roles provided greater access to alcohol at work because ‘technical’ spirits were used to clean equipment and could be misused for drinking (“*all that cleaning*, *they were all on spirits*”(#20)) and in other cases alcohol was offered as part payment (“*after fixing three machines they would give me 25g of spirit”* ((#12)). A few men thought that heavy drinking provided physical protection against their harsh working conditions (“*When I was working in the field the conditions were awful*. *We had [to drink] to keep warm (#10)”*, “*the radiation we get because of the job*, *we have to get rid of it somehow”(#4))*.

Several interviewees stated explicitly that the ‘*kollektiv*’ (a Soviet-era term for work brigade) they belonged to was crucial in determining drinking habits. Because Russian men’s socialising typically involves drinking, and work commonly provides the primary social identity for Russian men [44], a strong *kollektiv* especially in the early working life exposed them to many potential drinking occasions. Ritualised after-work drinking was typical, particularly on a Friday evening (“*of course*, *as we finish work*, *we drink (#4))* which sometimes took the form of organised binge drinking sessions: “*We celebrated birthdays*…*there would be 25 people*, *all putting money in*. *Our bosses let us …Sometimes I would wake up at home*, *somebody had had to bring me home” (#6)*. More everyday drinking occasions took place at the kiosks [small alcohol vendors] near work, at employees’ cafés, or on the journey home (“*they [kiosks] are all around the factory*, *they built them there specially*” *(#9))*. A minority described resisting pressure to drink heavily but non-conformity usually meant missing out on social events, which risked weakening of social relations and influence. Changes in the *kollektiv* and losing touch with old colleagues were associated with drinking declines, and these was described with nostalgia and regret because the men missed the camaraderie and friendship bonds drinking had created: “*We still go*, *but the group became smaller… and before the occasions were bigger*. *A big group of us would stay for longer for any reason*” (#9).

Over time employers’ anti-drinking policies had become stricter and directly restricted workplace drinking. In addition workers themselves felt an increased sense of responsibility and job insecurity compared with Soviet times when employment was taken for granted. Management monitored drinking more rigorously using more advanced technologies, and if caught drunk the penalties were more severe:

*“Well my work was more relaxed*, *when I would come smelling of alcohol they would just slap your wrist but now it’s all official*, *first they give you a warning and if you get caught for the second time you lose your job”*

*(#15)*

Sharing a bottle during the work shift was described nostalgically (“*in the good old days someone would come and offer a beer or two or and maybe some vodka” (#15*)).Although reduced since Soviet times, there were some contemporary accounts of workplace drinking which described lax anti-alcohol policies; in one example from a factory environment drinking sessions and even choice of the beverage were led by the manager *((I drink) vodka*, *because the manager doesn’t drink beer…so we all have to follow his example (#4))*.

#### Family influences

Reductions in heavy drinking often coincided with the men marrying, settling down and becoming fathers. These crucial life events marked a transition into a different life stage with a different set of behavioural expectations to conform to: performing the ‘breadwinner’ role demanded greater responsibility and resulted in less free time for drinking, less disposable income and family pressure to drink less. Whereas heavy drinking and bingeing were acceptable in youth they were incompatible with marriage and family.

Sometimes men felt coerced to reduce their drinking because family members monitored their habits (*my sons tell me off if I buy vodka from the shop*, *they check up on me” (#14)*, and a reoccurring theme was the controlling wife:

- *“Yes*, *there is less time now*, *and she is trying to control it*, *and she is calling me and trying to find out what I’m doing… we decide with our friends after work*, *to go drinking*, *and she starts calling me*, *so I only manage to drink a bit”*
*(#14)*
- *“So she is against you drinking completely*, *or against you drinking too much*, *which one*?*”*
- *“Just too much*, *so that I can walk home*, *do some chores round the house*, *and go to work normally”*

This social control of drinking within marriage [60] is consistent with other Russian studies [8,41]. On the other hand reducing drinking was sometimes perceived as a personal decision prompted by external events (“*My youngest daughter was born and after that things changed”(#21); “I met my girlfriend and that was a good stimulus for me to stop”(#12))*. The demands of spending more time with family and acting as a good role-model to their children (especially sons) superseded social pressure to drink with work mates or friends. In many cases this was not described as a conflict but accepted as a normal transition:

*“Before when I got my wages*, *I would hang around with my friends*, *but now I come back to her and talk about how to make it stretch…nothing has changed [in terms of quality of life]*, *just we are getting older and wiser*.*”*

*(#17)*

#### Ageing/maturing

The shift in emphasis from heavy drinking with workmates to more moderate drinking was mostly perceived as unremarkable, something that ‘just happened’, and part of the pattern of ageing: “*the age of 35–40…people above that age tend to drink less*, *from what I see*” (#1). Some described the craving to drink inexplicably lessening with age:

*“There was a sort of change*. *When I was about 30 years old*, *probably 35*, *there was a kind of tug*, *a pull*, *you understand*. *You go somewhere after work*? *So you drink…*. *And now*, *I simply don’t know how now it can be*, *just drinking and drinking*. *Well*, *sometimes I would hang out in company*, *and then I would say to them “If you want to- drink”*. *I don’t want to and that’s it*.*”*

*(#1)*

In other cases health concerns curtailed drinking (“*I loved to dance while drunk*. *Now it’s awful because I am not allowed to walk quickly” (#25))*. However several interviewees described cutting down on drinking as a conscious decision which originated from a desire to gain control over their habits and health (“*I am getting old now*, *and I want to live longer*” (#11)). Sometimes this was stimulated by seeing their heavy drinking friends getting ill or dying, or by noticing alcohol’s health effects on themselves (worse hangovers, for example). Having a sense of mastery over alcohol, of knowing your personal limits, was expressed with some confidence and pride: *“We know our dosage*, *we know our limit very well” (#23); “I am trying to behave myself in that respect now*. *Before I had less discipline” (#14)*. Car ownership, an important status signal for Russian men, was also described as a prohibiting factor, as they feared being caught drink-driving and thus losing this marker of mature social status.

### Increases, fluctuations and abstinence

Men who had these patterns tended to have more unstable employment and family histories than other interviewees, and stressful personal situations had triggered periods of heavy drinking:

*“I can tell you I started drinking in 1975*. *… That is the year my son was born disabled*.*”*

*(#7)*

*“My wife is constantly ill… She has invalidity and doesn’t work*. *They removed her bladder*, *for a long time she lived without going to the toilet*. *I began to drink more vodka*.*”*

*(#8)*

Dramatic fluctuations were sometimes associated with undergoing the alcohol treatment popularly known as coding (*kodirovaniye*), which usually refers to a suggestion-based method where the narcologist persuades the patient that they have altered their brain so that alcohol consumption is physically dangerous [37]. Coding was a temporary fix and believed to last typically for a few years. Some men were pressured to undergo treatment by family (“*my wife was nagging me about it”* (#24)) and others had feelings of personal failure inducing them to seek help. Some broke their coding and returned to heavy drinking as a way of coping with personal tragedy, accidents and stress. Those who successfully completed the period of abstinence under coding also returned to drinking afterwards.

Similar to the decreases described above, the ability to abstain without coding was often a source of masculine pride and self-esteem, a public display of willpower and self-control in the face of social pressure and addiction. For some abstinence was a personal challenge and a chance to earn the respect of relatives:

*“my wife’s girlfriend and her husband came over and they dared us that we couldn’t possibly quit drinking*, *and then we stood up the challenge*, *and for 2 years I didn’t touch alcohol…*

*(#17)*

## Discussion

The narratives presented here describe changes in drinking over a period of enormous social and economic instability. Within this context unexpectedly the most dominant drinking pattern trajectory in our study was a decline in heavy and hazardous drinking with age. Decreases were often attributed to ageing and ‘inevitable’ shifts in personal priorities over the lifetime from male bonding with colleagues in youth to performing the responsible breadwinner role for their wife and family. Drinking trajectories were heavily embedded in expectations of age, gender, and status-appropriate behaviour. The few men who had drinking increases or sharp fluctuations also tended to have more unconventional employment or family histories, and although it is difficult to know the direction of causality it is likely heavy drinking and social problems are mutually reinforcing [8].

Our study sheds light on alcohol use dynamics in Russia, which together with other Eastern European countries has the highest rate of alcohol-attributable deaths in the world, estimated to be 10 times higher than in other world regions [61]. Moreover, deaths disproportionately occur among middle-aged men [3] so an understanding of the social factors determining life course drinking patterns is crucial to the development of effective prevention strategies. Drinking patterns in our study were consistent with previous studies which show that mean male drinking peaks in young adulthood, plateaus in mid-life before declining in older age [62,63]. However by focussing on average effects these studies fail to identify different lifetime alcohol use trajectories and more importantly the social factors underlying them. Recent studies in the UK have highlighted the associations between family roles and reduced drinking in mid-life [64] and that perceptions of age and status-appropriate behaviour, and friendship maintenance are important determinants of male midlife drinking patterns [52,65]. Our study makes a unique contribution by exploring these issues within an extremely heavy drinking population.

Consistent with other studies this study showed that the construction and performance of Russian masculine identity is entwined with heavy drinking [8,38,44] and that heavy drinking in all-male groups (especially with work colleagues and in young adulthood) was seen as routine, expected behaviour. All-male drinking occasions had a social function (particularly among young men) because they fostered male camaraderie and identity, and strong social and work bonds, similar to contemporary accounts of railway workers’ drinking in Ukraine [10], and to Scottish men’s accounts of drinking and friendship [65]. Increased social participation is likely to have various (potentially offsetting) influences on health behaviours [66], but in the context of Eastern Europe male sociability appears associated with higher levels of alcohol use [67] driven by the norms of masculine behaviour. The narration of particularly raucous drinking sessions or ‘drinking tales’ [68] reflected a nostalgia for youth, Soviet era workplace bonds [21] and an assertion of masculinity. The all-male context of heavy drinking contrasts with Western European countries where drinking is more likely to take place in more gender-neutral contexts such as restaurants and the home [69].

In young adulthood the blue-collar workplace was a crucial site for the formation and reinforcement of male drinking norms, both through the cultural ‘habitus’ of the *kollektiv* and through more structural means such as availability and the institution’s alcohol policy. This was not only true of Soviet-era plants, but also for some contemporary industrial workplaces. In addition workplaces also influenced drinking directly through (non-)enforcement of the company alcohol policy, the availability of non-beverage alcohol at work, and the strategic positioning of alcohol kiosks around workplaces. These factors work together to create an ‘alcogenic’ environment [70]. Further research should be conducted in contemporary workplaces to understand the potential for public health initiatives in that environment to reduce hazardous drinking.

Life course reductions in drinking can be interpreted within the framework of social conformity to age-based masculine norms. Russian men assert their masculinity in different ways over their life course, which has implications for their drinking pattern. Young men are more likely to drink heavily and hazardously while socialising with their workmates which helps to develop the strong friendships and instrumental social networks important for securing stable employment and a reasonable standard of living. By contrast, as men reach their late 30s or middle age, they are expected to become reliable breadwinners and provide a good role model for their children [42], at which stage men usually reduce their drinking, either voluntarily or as a result of external pressure. For some older men exerting self-control over alcohol (and other habits such as smoking) was an alternative assertion of masculinity and contradicts studies portraying Russian men as passive with a weak sense of individuality [50]. Older men also spent more time at home where drinking opportunities are rarer, likely to be mixed sex, and featured more controlled drinking. The contrasting cases of men whose drinking did not decline tended to have disrupted family lives. The conformity to age and gender-specific social expectations can be seen as ‘doing gendered age’, a theory that has been applied to Russian female care-giving [53,54] but we argue extends to Russian men. This also provides a less static account of Russian masculinity and drinking than in previous studies [38,50].

The influence of age-based norms on Russian men’s drinking may become less important, however, in times of acute social stress and instability, particularly when employment and maintaining breadwinner status are threatened. The population-level increases in alcohol-related mortality in the economically unpredictable 1990s and at the time of the 1998 rouble crisis [28] and micro accounts on the interplay between employment and alcohol [8,21] suggest that many Russian men responded to social and economic instability by drinking more harmfully, irrespective of age. This is related to the central role of work and wage-earning in defining Russian men’s identity [44]. Moreover this implies that future social instability could produce further fluctuations in alcohol-related mortality.

Our study had some limitations. Many of the accounts of drinking are historical and further research is needed to understand alcohol in the contemporary Russian workplace. Despite purposive sampling we ended up with mostly accounts of drinking decreases. Whether this represents a survivor effect or selection bias is less important than our missed opportunity to explore increases in detail. Seven men had participated in an earlier motivational interviewing trial, and while this was not found to be significantly effective [57], it could have influenced their recent trajectories. We used a mixture of male and female interviewers which could have caused response bias. However we compared reports of recent alcohol consumption with those given in the formal longitudinal interviews at IFS-1 and IFS-2, but found no systematic reporting bias according to interviewer characteristics.

This study is unique in exploring detailed alcohol use trajectories in Russia, where men’s alcohol-related mortality is a serious public health issue. The results highlight the importance of blue-collar workplace socialising for conditioning patterns of hazardous drinking, but also that this could be a potentially successful intervention site. Further research should be conducted focussing on alcohol in a variety of contemporary workplaces, and to investigate the trajectories of men who weren’t in this sample due to survivorship or other issues. The study also highlights the importance of considering social conformity to both age-and gender norms for influencing health behaviours in Russia.

## Supporting Information

S1 Dataset Dataset of transcripts which support the study findings. (DOCX)
