# Supplementary material for: Social Factors Influencing Russian Male Alcohol Use over the Life Course: A Qualitative Study Investigating Age Based Social Norms, Masculinity, and Workplace Context
Source: PLoS One. 2015 Nov 17;10(11):e0142993. doi: 10.1371/journal.pone.0142993 (PMC4648522; doi:10.1371/journal.pone.0142993)
Supplement: S1 Dataset — (DOCX) [file pone.0142993.s001.docx]

**S1 dataset. Dataset of transcripts which support the study findings.** Includes important information and excerpts/quotes used in support our results (text in Russian and English)**.**

**Throughout- R= respondent; I= interviewer**

**Interview#1. Decrease.**

**Russian version: Excerpt 1**

R: ну пытался. Не получается пока. Все равно надо будет, хотим мы этого – не хотим, придется, надо. А с выпивкой, с выпивкой, че с выпивкой. Можно выпить – можно не выпить. Последний раз я пл, наверное, 1 мая, что ли.

I: так давно?

R: да мне вот как-то, понимаете… ну вина я, обычно, как его..вино испанское, болгарское – вот эти вина вот есть..

I: угу, сухие, да?

R: да, ну они… как они…9-12 градусов.. водку я не пью… так..пиво я вообще…я уже наверное, больше лет 10, а может и больше не пил. Я не представляю, как это пиво пьют люди.

I: угу

R: я бы лучше вылил.. ну пиво, я не понимаю как, для чего это пиво нужно

I: я, кстати, я тоже даже запах не переношу

R: нет, во-первых, запах и… ну я не знаю, всякие люди, ну я говорю, все мы разные, все мы разные.

I: угу, а вот это вот.. вот Вы всегда так равнодушны к алкоголю были всю жизнь или какие-то периоды?

R: нет, были, да. Было тяга в одно время. Щас скажу. Это мне было где-то…ну лет 30, наверное, 35 вот было… ну не то ли что тяга была, не то ли что тяга, да вот понимаете… Выйдешь после работы куда? Вроде давайте выпьем. Один – выпьем, второй – выпьем..

I: А Вы на этом же заводе работали?

R: Нет, на [factory in Izhevsk] я работал в то время

I: Ммм..

R: и тут видите, нуу вот че.. от коллектива и режим сам по себе много значит. Я вот сам по себе понял так, что по тем временам, я вспоминаю, вот в это время вспоминаю… для себя вот выводы сделал: чем больше пьешь, тем больше хочется. Вот выпьешь, вот вроде бы как пятница, как пятница или там определенный какой-то день… надо бы маленько выпить… А щас, просто видите я не знаю как щас как-то что есть, что нет.. ну выпью и выпью

I: привычка не сформировалась?

R: ну да, в компании ходили, че там..сюда вот приходят, я говорю «хотите – пейте», я говорю . Не хочу просто и все. Не то ли вот что-то..ну просто не хочу, и все ..

**English: Excerpt 1**

R; I tried. Didn’t manage yet. It doesn’t matter .It’s not about wanting, it’s about needing. And with drinking, with drinking, with drink. I can drink, or not drink. The last time I drank was probably May 1st..

I: That long ago?

R: Yes for me, it’s like this, understand, I drink wine, Spanish wine, Bulgarian, these kinds of wine

I: Yes, dry, right?

R: yes, about 9-12 %, I don’t drink vodka, Generally I don’t drink beer, I also probably, more than 10 years, or more I did not drink. I don’t understand, how people drink beer.

I: Aha

R: I would drink better... but beer, I don’t understand why people need to drink this beer.

I: By the way, I can’t stand the smell

R: No, firstly, it’s the smell, I don’t know, any people, they say, we are all different, we are all different

I: Aha- yes that right, it’s like that. You always had such a take it or leave it attitude towards alcohol your whole life or just in this particular period?

R: No, was a sort of change one time. When I was about 30 years old, probably 35 I was, there was a kind of tug, a pull, you understand. You go somewhere after work? So you drink. Once- we drink, twice—we drink

R: And in which factory were you working?

I: In this time I worked at [factory in Izhevsk] .

R: - Mmm

I: You can see here, from the work collective many people know it. I remember myself that in these times, in these times I remember, You can make the conclusions for yourself, you drink more, then wants to drink more. You drink, you drink again like on Friday or a certain day, you need to drink a little. And now, I simply don’t know how now it can be, just drinking and drinking

R: You didn’t form a drinking habit?

I: Well, I did hang out in company, and then I would say to them “ If you want to- drink”. I don’t want to and that’s it. And I simply don’t want, and that’s it.

**Russian: Excerpt 2:**

R: мне кажется, все ж таки ни компания, ни работа ,а вот для меня вот, я для себя вот вывод сделал так: я вот выпивал, да, я начал болеть, почувствовал, что начал болеть, утром встаешь, вот очень тяжело встаешь, и лучше я не буду пить, чем вот чтобы так тяжело было

I: т.е. все-таки на здоровье почувствовали?

R: да..да…хмель, вот это похмелье все, недомогания. Я думаю, лучше я… И вот сейчас вот в настоящее время, говорю вот чем меньше вот… вроде бы и это… нормально жить, нормально общаться можно и с такими, кто пьет…

R: Т.е. получилось, что… Ну тем не менее, у Вас изменилось вот само окружение после того…?

I: нет. То же окружение, те же друзья остались, те же друзья вот здесь приходят те же друзья на (17.04), и там, дома. Вроде бы ниче такого не изменилось. А окружение по работе – люди меняются конечно, люди меняются, но…ну тут видите как.. вот ваша организация, предположим,да,…. все зависит, многое зависит от самого руководителя. И в каждом…в каждом коллективе вот..свое как-то вот…внутреннее вот…я не знаю, как…в каждый коллектив вот другой коль я вот в одном…ну я много… не много мест конечно поменял за свою трудовую… но я вот с железной дорогой частично маленько работал…это был…когда…дефолт-то вот этот начинался.. не то что вот этот, а тот еще, первый, В 90-е..в начале 90-х годов. Я вот в одном коллективе пришел..там совсем другое: и там по-другому поставлено, и руководство тоже по-другому. Там было по-другому, тут по-другому, тут по-другому. Не знаю, мне кажется, а что оно должно влиять…

R: ну т.е. получается, что если руководитель, допустим, не пьющий и как-то к этому относится отрицательно, то и коллектив не пьет? Вот правильно я Вас понимаю?

I: ну не знаю. Ну вот у нас тоже, тоже пьют многие, и пьют безбожно и на работе, и выгоняют с одного места… и руководитель, бывает, пьет. Ну мне кажется, все равно от себя самого зависит, будет он пить или не будет он пить

**English: Excerpt 2**

R: No. My circle, all the same friends, so nothing changed. Circumstances at work, the people are changing, obviously, they always do, but here is the situation. So your organisation suggested it all depends on management itself. And in each, in each workplace , ah it goes, inner , I don’t know how, in each working environment there is something, it’s too much for me, I didn’t change many workplaces in my life. On the railways I worked for a little while, it was when the default started, not that time, but the first time, in the beginning of the 1990s, so I worked in a completely different workplace, and it was completely different to my current one, even the management was different, everything was different. I am not sure but it looks to me that this thing could be influenced by something.

I: So if management, for example, don’t drink, and don’t approve it, so nobody drinks at that place? I understand you correctly?

R: I don’t know. So with us there are too many people drinking, way too many, and so they lose their job, and so the management happens to be drinking as well, but still it depends on oneself, and one’s own decision whether to start drinking or not.

**Interview #2: Decrease**

**Excerpt 1: Russian**

I: а вот всегда Вы одинаково употребляете, или у Вас в жизни были периоды, когда Вы пили больше, или, наоборот прекращали?

R: больше пил в Феодосии...когда служил в[*Crimea*]..после Афгана

I: Вы в Афганистане воевали?

R: и вот там, после, когда служил, пил

I: много, да?

R: ну, не много, но больше

I: тогда у Вас уже семья была или не было?

R: Да, была уже

I: вместе с Вами там жили?

R: угу

I: ну а уменьшили-то когда, в какое время?

R: здесь уже пил меньше

I: там..через полгода примерно

**Excerpt 1: English**

I: But in your life, were there periods when you drank more, or less? Or you quit at some point?

R: I drank much more when I was in [*Crimea*] , I was serving there, after Afghanistan

I: So you were serving in army during Afghan war?

R: Yes, when I was fighting in Afghan war, I was drinking

I: A lot?

R: No, not a lot, but more than usual

I: You already had a family by then?

R: Yes, I had

I: You lived together?

R: Yes,

I: So when exactly did you decrease your drinking?

R: I started drinking less here. So, after about 6 months

**Excerpt 2: Russian**

I: а вот когда Вы работали здесь в 90-е, как у Вас с алкоголем было? Употребляли как сейсас, или больше, или меньше?

R: чуть-чуть побольше

I: всё же больше, да?

R: то есть с возрастом Вы стали меньше употреблять?

I: а это с чем связано – уже потребности нет, или всё-таки сами себя как-то ограничиваете?

R: сам..

I: то есть считаете, что не надо много, да?

R: да

I: понятно. А вообще-то когда-то так бывало, что сам себе говорили – всё, я не буде больше?

R: нет

I: и сейчас таких мыслей не возникает?

R: нет

I: а Вам это для чего?

R: (смеётся) так спросите это у каждого мужчины

I: а мы у каждого и спрашиваем, все по-разному говорят, у каждого свои мотивы

R: нуу, типа с друзьями, отдохнуть

I: то есть Вы с друзьями выпиваете, да?

R: да, что я один что ли пил?

I: а друзья по работе или соседи?

R: даа, у меня и в саду есть, и по своей работе иногда встречаются,

I: но, в принципе, раз Вы водите за рулём, всё время-то не будешь пить..

**Except 2: English**

I: and were you working here in the 1990s, how was your situation with alcohol? You were drinking more or less?

R: Slightly more than now,

I: slightly more, yeah? So it means that as you got older you drank less? What was it connected to- you just don’t feel like it, or you tried to reduce?

R: I tried to reduce it myself

I: So you think that you shouldn’t drink that much. Right?

R: Yeah

I: Understood. So when you are drinking, do you say to yourself, enough, I won’t drink anymore?

R: No,

I: and you are not thinking about it now?

R: No

I: what do you need this information for? (laughing) are you asking every man about this?

R: Yes, we are asking every man, and every one gives different reasons

I: My reason being that I enjoy drinking with my friends

R: So it means you are drinking with friends, yeah?

I: Well, you don’t expect me to drink alone, right?

R: These friends, are they from work, or neighbours?

I: Yes, I know one neighbour, and sometimes guys from work

**Interview #3: Decrease**

**Excerpt 1: Russian**

I: были ли у Вас в жизни, когда Вы пили очень много? Ну..на Ваш вхгляд..

R: сколько много?

I: ну, чтоб Вам казалось, что это чересчур..Такое было?

R: ...было..

I: можете рассказать? Когда это? В связи с чем было?

R: ну когда это было..(длинная пауза) ..не знаю...даты не помню..

I: ну, не обязательно точно, а вот ..может, это с к4аими-то событиями было связано, какие-то обстоятельства..

R: ну было..собираемся с друзьями..накушаемся..

I: то есть это бывает не то, чтобы в какие-то периоды жизни, а бывает – иногда и сейчас?

R: нет, сейчас нет..не велено..не в моём положении

I: сейчас совсем не пьёте? Ни грамма алкоголя, да?

R: (отрицательно качает головой)

**Excerpt 2: Russian**

R: ну, попил какой-то период, потом всё..

I: то есть – попил каждый день? ТО есть у всех это по-разному, поэтому спрашиваю..

R: ну я и говорю – попью сколько-то дней, потом – всё..отдыхаю..

I: сколько дней отдыхаете?

R: ну, может десять, может, две недели..Потом случай какой-нибудь – опять..

I: а вот случай – это когда какая-то компания? Друзья собираются? Или какие-то другие случае бывают7

R: ну, позовут друзья в сауну....вот, охота пожариться..ради Бога – заходите..ну – пожарились, попили.

I: пили-то сильно? Или нормально до дому добирались?

R: сильно..когда у нас слабо-то было? Раз попало – значит..

I: но не обращались куда-то? Не было такого, чтобы из запоев выводить? Или было?

R: да нет..я..обходился..

I: ругали дома-то?

R: да конееечно..как же без этого..

I: а вот Вы говорите – с друзьями..Друзья по работе в основном были?

R: и не по работе..

I: а вот на работе Вы выпивали..прямо на работе?

R: ну..выпивал..

I: на это смотрели сквозь пальцы?

R: нет

I: тоже ругали?

R: конечно..ну так..изрека бывало..

**Excerpt 1: English**

I: and were there periods in your life when you drank too much?

R: How much is too much?

I: So what do you think is too much? Did it happen?

R: It happened

I: Can you tell me , ,when and why?

R: It happened (long pause) I don’t know, I don’t remember the dates

I: I don’t need all the dates, but maybe was connected to some events

R: Oh yeah, sometimes you get together with your friends, get drunk,

I: Oh yeah, did it happen, at some periods, what is more or less, now or then?

R: No, not yet, not now, not in my position, not in situation

I: so now you don’t drink at all? Not a single drop?

R: (he shakes his head)

**Excerpt 2: English**

R: Well I was drinking at some point, then stopped

I: Meaning you drank every day, I mean, everybody got a different situation, thats why I am asking you

I: And so I say, I would drink for a few days, then give it a rest

R: How many days of rest?

I: Of, at least 10 day, 2 weeks, but them something happened, and I started drinking again,

R: but what could possibly happen? Gathering of friends? Or some other event?

I: Yes, well friends gathering in sauna, sometimes you get a feeling that you want to go to the sauna, but then it leads to you getting hot, then getting drunk,

R: Getting really drunk? You were still able to get home by yourself?

I: Of yeah, getting really smashed, we never used to take it easy,

R: Have you ever seen anyone about it ( like a narcologist? Have you ever seen specialist, who would help you to get out of zapoi?

I: No, I managed myself

R: Did you have family conflict ( were they angry with you) because of that?

I: Yes, of course, that’s an unavoidable part of it

R: You said, you were together with friends, work friends?

I: Half from work, half from outside

R: So it means that you were drinking at work too?

I: I was drinking at work

R: And did you get away with it?

I: No, they were quite strict.

**Interview #4, classified as decrease**

**Excerpt 1: Russian**

**R** : Да нет, ну как бы меньше, реже стал пить.

**I:** Реже.

**R:** Да.

**I:** А с чем это связано, нам очень интересно, почему люди меньше или больше.

**R:** Ну семью завел.

**I:** То есть вот когда женились, да.

**R:** Да.

**I:** А это было, ну как бы жена что ли, она вам, вас просила, ограничивала.

**R:** Да нет, кА бы раньше на улице с мужиками туда-сюда, дома то тогда никто не ждал еще.

**I:** Угу. То есть просто заполнять свободное время не чем.

**R:** Ну да.

**I:** А в каком вы сошлись с женой?

**R:** В 2006 году.

**I:** В 2006 году, да? И с тех пор просто у вас получилось, что у вас времени на это.

**R:** Да мало, да и контролирует, звонит что, куда, где ты там.

**I:** Контролирует?

**R:** Да. Мы с мужиками решим там после работы, все, уже звонит, конкретно уже, по многу не выпьешь.

**I:** То есть она против того чтобы пил. Она против того чтобы вообще пил или чтобы в меру. Как?

**R:** Ну чтобы в меру, нормально приходил, дома шевелился, работал.

**I:** Угу.

**R:** Не просто пришел, упал. Уснул.

**Excerpt 1: English**

R: yes, of course, I started to drink less, and not so often

I: Not so often?

R: Yes

I: Is this connected with anything? We are very interested in why people start drinking more or less...

R: Well I have started a family now

I: Meaning that you got married?

R: yes

I: Did it ever happen that your wife insisted that you shouldn’t drink that much?

R: Before the marriage, I had more time to spend with my friends drinking.

I: Meaning that this is how you passed your free time, because of nothing else?

R: Yes

I: What year did you start the relationship with your wife?

R: In 2006

I: So from 2006, you didn’t have time for it?

R: Yes, there is less time now, and she is trying to control it, and she is calling me and trying to find out what I’m doing

I: So she is trying to control you?

R: Yes, we decide with our friends after work, to go drinking, and she starts calling me , so I only manage to drink a bit

I: So she is against you drinking completely, or against you drinking too much, which one?

R: Just too much, so that I can walk home, do some chores round the house, and work normally

I: Yes

R: Not just not to come home and to fall asleep straight away

**Excerpt 2: Russian**

**R:** То есть не наблюдали, с чем это может быть связано, переутомились или наоборот время есть там.

**I:** Да нет просто так, ну охота иногда и все, желание есть просто, организм требует.

**R:** Организм требует (смех). Все-таки организм требует, не то, что там душа или компания.

**I:** Да, организм.

**R:** То есть не за компанию, а чисто ваше желание такое вот, да.

**I:** Ну за компанию, один то я не пью никогда.

**R:** А вот на работе у вас народ пьет?

**I:** Пьет, как не пьет.

**R:** Ну вы не вместе там.

**I:** Как вместе, там тоже выходим иногда пьем после работы.

**R:** Ну там пиво, наверное, или тоже водку?

**I:** Водку, у нас как бы мастер не пьет пиво и я, говорит не пью категорически пиво не пью. И все мы под мастера.

**R:** Дак, вы во главе с мастером пьете, да?

**I:** Ну.

**R:** Ага, но жена ругается, если вы где-то там пьете.

**I:** Но как, у всех ругается так.

**R:** Ясно, а вообще то там у вас ни какой борьбы с алкоголем на предприятии.

**I:** А, что, чем бороться то, ну будем пить, так и будем пить.

**Excerpt 2: English**

R: Not connected to anything, this desire just comes and if my body demands it

I: (starts laughing) your body demands it...not your soul, not the company you keep

R: Yeah, my body demands it .

I: So it’s not like a social thing then? It’s your desire that suddenly comes

R: No, I have to have friends around, I don’t drink by myself

I: So your colleagues drink too?

R: Of course they do

I: So do you drink all together?

R: Yes, of course, as we finish work, we drink,

I: Is that beer or vodka you drink?

R: Vodka, because the manager doesn’t drink beer, I don’t drink it on principle, so we all have to follow his example,

I: So you are drinking together with your manager, all of you?

R: yes

I: But your wife, she starts a scandal( she objects) when you are somewhere else drinking?

R: Yes, of course, they all do because of that , all other men they have the same problem

I: Understood, so it means that at your workplace, there is no anti-alcohol policy at all?

R: Why would anyone have an anti alcohol policy? We feel like drinking and we will drink..

**Interview #5: Decrease**

Excerpt 1: Russian

I: всё-таки как изменилось отношение к алкоголю? Качество? Количество?

R: Количество, конечно.. и качество ну, может, в молодости – в 60-70 годы – портвейнчику с ребятам...в студенческие времена...а сейчас – водку если я пью, то качественную...эти всякие «Удмуртии» и «Калашниковы» не беру...Для себя же. Ну а бывало – и одеколончик пробовал в армии...А потом - нет

I: Были периоды, когда Вы вообще не пили, нисколько?

R: было. Хотел – и не пил

I: сколько примерно это могло продолжаться?

R: ну вот как-то я полгода или даже год вообще не пил

I: а в связи с чем возникал такие желания?

R: ну вот так. Вот напился! Плохо! Говорю себе – какая я сволочь! Всё! И всё..были такие моменты, да...в основном, в компании...один-то я - не так...ну смотря какая компания...обычно, один – всё в меру, нормально. А вот если встретились где-то, да зашли в какую Пирожковую, о – ё-маё...или ..есть у меня друзья...правда, они падают чуть быстрее...

Excerpt 1: English

R: Quantity, of course! But quality I remember when I was young in 1960s and 70s we were drinking port when we were students, but now I tend to drink more vodka, but if I buy it I buy the best quality I can find, vodka is like Udmurtia, and Kalashnikov, don’t even look at them, why would I choose rubbish for myself, when I was in the army we tried some eau du cologne but after that, never

I: So you had some periods where you didn’t drink at all?

R: Oh yes, I had these periods, I didn’t feel like it.

I: So how long did these periods last?

R: Sometimes they lasted 6 months, sometimes even 1 year

I: So why did you have this motivation to stop?

R: Well sometimes I would drink too much and feel guilty about it, so I said to myself, fine, stop it, these moments they were happening, but after a gathering of friends drinking, but when I’m drinking my myself, I don’t reach this stage at all, but when we meet with friends, once we start partying, we never can stop, but good thing that my friends get drunk faster than me

**Interview #6: Decrease**

Excerpt 1: Russian

R: Нет, не было таких периодов. Бывало, когда работал на старой работе. Дни рождения отмечали на работе. Скидывались по 100 рублей. Тогда ничего получалось на 100 рублей. На эти деньги стол накрывали. У нас всегда это проходило по пятницам. И столовая была как комната отдыха. 25 человек рабочих было, скидывались. Из начальства никто не запрещал в пятницу остаться выпить после работы. Чтобы напиваться не бывало. Но бывало пару раз, что переберешь, проснешься дома. Доставят до дома. Тогда не больно много кто пил. У всех машины были. По тем временам заработок хороший был.

I: А сейчас на этой работе скидываетесь?

R: Сейчас директор денег дает и в пятницу стол накрываешь на 7 человек. Торт, чай. Если есть желание, то можно спиртное занести.

Excerpt 1: English

I: was there a period when you drank more heavily?

R: No there wasn’t really such a period. Sometimes when I worked in my old job and we would celebrate someone’s birthday. We would all put 100 roubles on the table, the table would be covered with notes. This would always happen on a Friday. The canteen was like a recreation room, there would be 25 people there, all putting money in. Our bosses allowed us to drink on a Friday after work. Sometimes I would wake up at home, somebody had to bring**/**take/drive **me** home. Those days not a lot of people drank. [as] Many had cars, at that time we had good salaries.

I: and do you drink after work now (all put money in)?

R: Now the Director puts in money for 7 people to have tea and cakes on a Friday. If you want, you can take a shot or so of vodka. If you want you could bring alcohol

**Excerpt 1: Russian**

R: да, выпивать, конечно, особенно в начале 90-х пиво, водка. Практически не то, что мы пили пиво а так. Бывало, что и дни рождения, все это хозяйство отмечали.

I: Больше пили пиво или водку?

R – По большей мере, наверное, пиво употреблял.

I – Тогда было тоже такое разнообразие пива или нет?

R – Нет, тогда только-только появлялось в банках пиво, баночное пиво и 350 граммов, по-моему тогда маленькие баночки еще были. Вот это я хорошо помню.

I – Вам было просто любопытно?

Р – Да нет, употреблял, на днях рождения – водка, так иногда вечерами вот пиво, но шибко не злоупотреблял. К алкоголю у меня нету такой тяги. Потом вообще не стал водку пить.

I – А после армии как?

R – После армии что приходилось, бывало, что соберемся с друзьями, тогда еще жил на *[street name*] (С родителями – прим.Дементьевой), в неделю раз-то по выходным бывало, что пировали.

**Excerpt 2: English**

R: Oh yeah that happened quite often at the start of 1990s, we are talking about beer and vodka, it’s not like we were drinking for the sake of it, we were celebrating things like birthday, or house warming parties

I: So you were drinking mostly vodka or beer

R: At that point it was definitely mostly beer

I: So you were drinking beer of different kinds/brands?

R: No, that was the time when we started to have canned beer, and I remember it very well these 350 ml cans, this is what remember very clearly

I: So you were curious about these new beers?

R: No no I mean I was drinking not that much. Like on celebrations we would drink vodka, in the evening I would drink beer, but It wasn’t an addiction, I don’t have any addiction to alcohol, and then later on I quit vodka altogether

I: and after the army?

R: after the army we drank whatever we had, we gathered together, we lived in *[street name*], so once a week on the weekend we would get together and party

**Interview #7: Fluctuations**

**Excerpt 1: Russian**

I: но вот 75-й год, Вы говорите..тогда и пить начали, да?

R: вот..он и родился..

I: это младший сын?

R: нет, это старший сын..да..старший..потом..он – с 75-го..потом..в 76-й год у меня родился другой сын..вот щас....вот..

I: ну и что – много тогда пили, нет?

R: да нееет..я ж....много не пил я...ну там может..пол.очки (?)..дети..ну..сами знаете..немножко пил..

I: ну там, как-то без запоев было, да?

R: не, без..без-без...

I: ну..и..а потом..

R: а потом...резко я начал..нуу..здорово певый раз..когда..с женой мы разошлись...ну, она...

I: а это когда было?

R:..(долгая пауза)...ну...уф... у ..дочь...щас..она уже кончила..вспомню..как не соврать-то..

I: ну примерно хотя бы...

R: ну примерно она во второй класс пошла...во второй класс..ну там..она пришла..чё-то разругались..я говорю – а чё, говорю, сопли-то морщишь...Если ты живёшь с другим – живи..ну, через день она приходить начала...я говорю – иди, говорю, иди отсюда...и живи. Всё. Иди отсюда. Она .(?)...дочку забрала и ушла..Всё, с тех пор мы..вот у меня..11 лет...дочь..ну её щас 20 лет...

I: Ну, то есть..получается, что Вы лет 9 живёте уже без жены?

R: 9-10 лет..где-то так...

I:а когда с женой-то расстались, больше стали пить, да?

R: ну..ну а потом вот..разозлился, думаю..ну вот..чё-то..сперва..ну как..

I: обидно?

R: обидно было..ну и всё, и вот..начал-начал-начал...

**Excerpt 1: English**

I: That was 1975 you said, and you started drinking then

R: Yes, that’s when he was born

I: This is your younger son

R: No this is elder son, the next year in 1976, the next son was born

I: So you were drinking a lot then

R: Not really, didn’t drink much, well you understand I was drinking a bit,

I: So it wasn’t like regularly

R: and then I started off quite seriously, and this is when I separated with my wife

I: and when was that?

R: oh well let me think about it, so I can give you the exact date

I: can you say approximately

R: approximately it was when our daughter went to 2^nd^ grade, and then my wife came and I said to her, don’t cry, just tell me if you are living with someone else, just tell me, she kept coming, but I sent her away , she came back and took the daughter with her, since then that would have been 11 years ago, because our daughter is now 20, and she

I: so you haven’t lived with your wife for at least 9 years?

R: yes, 9-10 years, something like that

I: When you split up with your wife, you increased your alcohol intake right?

R: Well well and after that I was really angry with her

I: were you upset

R: I was upset, and then I started ( drinking)

**Interview #8: Increase**

**Excerpt 1: Russian**

R: Жена болеет постоянно. В 1998 году операцию сделали. В последнее время часто болеет, особенно зимой. Летом еще есть какое-то занятие, на огороде, а зимой делать нечего. У нее инвалидность. Не работает. Удалили мочевой пузырь. Долго не может без туалета. Больше водки пить стал.

I: Какая причина?

R: Не знаю. Больше ленивый стал. По хозяйству чего-то делать нет особого желания. Сейчас ремонт затеяли.

I: А больше стали пить на работе, с друзьями или дома?

R: Дома в основном. На работе тоже иногда бывает.

I: А на работе?

R:Не просто так. У кого-то праздник или день рождения.

I: Но ведь раньше тоже были праздники. Сейчас почему стали больше пить?

R: В одно время контролировать сильнее стали. Потом перестали. Сейчас опять контроля жесткого нет.

I: А какой контроль, медкомиссия?

R: Нет у нас медкомиссии. У нас охрана частенько ездила и фиксировала.

I: То есть они следили что бы ничего не произошло,. чтобы все трезвые были?

R: Да.

I: Но у вас ведь травмироваться можно?

R: Да. Механизмы, электричество.

I: А почему сейчас они не стали контролировать ту же самую пьянку?

R: Не знаю. Народ держаться стал за свое рабочее место. Воля. Уже никуда не устроишься.

I: А раньше, когда они жестко контролировали, это какие года были?

R: Не могу сказать.

I: Ну, 3 - 4 года назад?

R: Пожалуй, да. П последний год как-то не так. Раньше постоянно в вагончик заходили в рабочее время или вечером. Заходили, проверяли, а сейчас по улице только ездят, по дорогам, где бы чего бы не уперли.

I: За пьяными им специально команду дают следить?

R: Может быть. Выпьешь маленько, так не видно, просто запах есть. Чтобы напиться и шататься - такого не было.

I: А бригадир за этим не следит, только служба безопасности?

R: А что он, мастер у нас в бригаде почти не бывает, все время в конторе. Бумагами завалили. Раньше мастер постоянно на участке был. Я когда мастером работал, все время был.

I: А на месторождении есть свой магазин?

R: Только буфет и столовая.

I: А где народ берет выпивку?

R: С собой привозят. Бутылочку взял, привез.

I: Но в принципе, на работе выпить могут?

R: Да, маленько.

**Excerpt 1: English**

I: what else has happened?

R: My wife is constantly ill. In 1998 she had an operation, in the last few years she was always ill, especially in the winter. In summer there is something to do in the garden, but in winter she does nothing. She has invalidity and doesn’t work. They removed her bladder, for a long time she lived without going to the toilet. I began to drink more vodka.

I: for what reason?

R: I don’t know. I became a bit more lazy, I don’t have a particular desire to do anything around the house, now I started to do some repairs

I: and did you start to drink more with your friends at work or at home?

R: Mainly at home, but sometimes at work

I: and at work?

R: not so much as at home, maybe if there is a holiday or birthday celebration.

I: But before there were also celebrations. Why did you start to drink more now?

R: At one time it was strongly controlled. Then it stopped. Now there are no strict controls over drinking alcohol.

I: and what kind of controls were they, a medical committee?

R: no we didn’t have a medical committee. We had guards coming round and making notes.

I: so they came round and checked, and nothing would happen, if everybody were sober?

R: Yes

I: but if you drink there is the risk of getting hurt?

R: Yes, there are machines and electronic parts

I: and why these days they don’t control drunkenness to the same extent?

R: I don’t know. People now became more careful about about keeping their job. Freedom. There’s nowhere to find a new job.

I: before, when it was more strictly controlled, when was that?

R: I can’t really say

I: well, like 3 or 4 years ago?

R: Probably, yes. In the last few years it wasn’t like before. Before they would constantly come in the van to the workplace, it could be in the morning or evening. They used to come in and check you. Now they only go in the street, where you are not likely to bump into them.

I: Do they give special instructions to monitor the drinkers?

R: Maybe. If you drink just a bit, it won’t be noticed because there is no smell. If you get drunk and stagger, this is not OK.

I: and the foreman doesn’t watch you, if only for security?

R: Well our supervisor of the brigade is almost never around, he is in the office all the time, doing paperwork. Before the supervisor was always in the workshop, that was when they used to work all the time.

I: and it has its own shop on the factory complex?
R: there is just a cafe and a canteen

I: so where do people get alcohol from?

R: they bring it themselves, a bottle or so

I: OK so in principle, you can drink at work?

R: yes, a little

**Interview #9: Decrease**

**Excerpt 1: Russian**

R: Тут как, раньше пиво, может, больше пил лет 5 назад, а сейчас уже как-то... Зимой вообще не пью сейчас. А так только водочку.

I: А пиво после работы или компанией собирались?

R: Обычно после работы кружечку выпьешь.

I: А у вас там есть где?

R: Сейчас везде ларьки есть. Ну, так, не куда попало едешь.

I: Если бутылочку - то везде, а если кружечку, то в рюмочной.

R: Их полно вокруг завода. Специально они и построены там. Везде они. Если мельком посмотреть, то они около [ names of factories in Izhevsk], ну, депо еще там рядом. Там кругом они.

I: И вы после работы заходили?

R: Туда редко. Может в месяц раза два. Когда как получается. Бывают праздники разные, корпоративы, юбилеи. Но юбилеи обычно в кафе. Юбилеи получше уже. Не как в какой-то забегаловке. Бывает, зайдешь мимоходом, с бригадой, с товарищами. Ветераны приходят повидаться. Идешь, посидишь где получше.

I: А потом что произошло? Просто перестали ходить?

R: Нет. Ходим, как же. Просто народу стало меньше. Своего окружения уже мало. Все одни пацаны. Раньше чаще ходили. Бригадой челевек 10 соберется. Празник там...

I: Больше человек, больше поводов.

R: Да. И поводов больше. Большая компания дольше сидит в любом случае. А тут 2-3 человека. Они сели, посидели полчаса максимум. Встали и ушли.

I: Это вы сейчас про пиво говорите?

R: Нет. Пиво я так при коммунизме пил до 90-го года, когда его не было. Вот тогда добрался до какого-нибудь пивбара. в городе их можно было пересчитать по пальцам. Нормальных было 1-2, а таких забегаловок как на [main street in Izhevsk]... Туда я и не ходил. А сейчас разливное, живое пил. В этом году даже еще и не пробовал. Не тянет. Есть люди, пьют каждый день. В мороз и всегда у столика. После работы люди выходят, на остановке есть ларек.

I: Бывает, что сразу компания ходит.

R: Сейчас так не ходят. Пацаны что они, в бригаде пара работает более-менее.

I: У вас слишком большая разница в возрасте и вы с ними не ходите?

R: Нет.

I: А водка?

R: У меня один человек в бригаде остался с которым я более-менее. С других бригад еще попадают. На праздники бывает, что собираемся вот, буквально в последний раз, человек 5 максимум. Иногда приходят еще старые работяги, которые уже не работают, по старой памяти. Максимум 4-5 человек собираемся.

**Excerpt 1: English**

R: Well, in the past I drank beer, I drank more maybe 5 years ago, and now hardly anything, In the winter I don’t usually drink, maybe only a shot of vodka

I: and did you drink beer with your mates after work?

R: Usually after work you have a drink.

I: and where do you go?

R: there are stalls everywhere, you go wherever they want to go.

I: If it’s a bottle- that’s everywhere, but if it’s a pint, or a shot of vodka

R: they are all around the factory, they built them specially there, everywhere. If you take a look, they are all around [ names of factories in Izhevsk], well still around the depot.

I: and after work you stop by there?

R: Now it’s more rarely, maybe twice a month, when I can. There are various holidays, corporate parties, jubilees. But for jubilees it’s normally in a cafe, they are better, not like in at a stall. Sometimes you go in passing with your work mates, and veterans drop by to see you. You go where you can get a good place to sit.

I: so what happened after that? You just stopped going?

R: no we still go, but the group became smaller, my circle of friends is quite small, sometimes I am all alone, before I went more often, there was a group of 10 people or so, we had celebrations...

I: more people, more occasions

R: Yes. And the occasions were bigger. A big group of us would stay for longer for any reason. And now its 2 or 3 people maximum. They sit and stay for half an hour maximum, then stand and leave

I: and are you now talking about drinking beer?

R: No. I drank beer during communism until the 90s when there was none around. We would go to some pub-bar, you could count them on one hand. Normally there were 1 or 2 and snack bars like like at [main street in Izhevsk] , I didn’t go. And now they have live beer on tap, to his day I still haven’t tried it, it doesn’t attract me. There are people who drink every day, in the cold and always with dinner. After work these people leave and there is a beer stand at the bus stop.

I: you go only when your mates go?

R: now I don’t go. Guys who do that work more or less in pairs together

I: so there is too much of an age difference and that’s why you don’t go?

R: No

I: and what about vodka?

R: In my team only one person stayed with me. Some are still in other teams. On holidays sometimes we get together, on the last time there were literally 5 people maximum. Sometimes former workers join us, those who don’t work anymore. Maximum 4 or 5 people.

**Interview #10: Decrease**

Excerpt 1: Russian

R: Употребляю аккуратненько.

I: А 5-7 лет назад в таком же количестве?

R: Нет, поменьше.

I: А с чем это связано?

R: Женился, жена, ребенок... Времени нет.

I: А раньше как, на работе, с друзьями встречались?

R: Да, на работе. Раньше работа в полях была, тяжелые условия. Приходилось чтоб согреться. Ну, это так, тоже праздники. Когда 100 человек мужиков в одном котле варятся, то психологически тяжело. Там еще погодные условия тяжелые.

Excerpt 1: English

R: My drinking is all under control

I: and you drink more than 5 7 years ago?

R: No, a bit less.

I: and why is this?

R: I got married, my wife, child, no time

I: and before, did you used to meet with friends at work?

R: Yes, at work. Before work in the fields it was awful conditions. We had to drink to keep warm, and of course there were celebrations. When there are 100 men together in the same melting pot, its psychologically difficult. And the weather was awful.

**Interview #11: Fluctuations**

**Excerpt 1: Russian**

**R:** С похмелья пообещал семье, что пить брошу.

**I** – Ну, а семья как бы до этого просила или.

**R** – Нет, ну, просто это у меня таких запоев не бывает, ну я допустим один раз напьюсь и там достаточно. А тут, что-то у нас получилось 3 дня рождения заподряд на работе. Ну и короче это, был с глубокого похмелья и сказал, что с 3 мая брошу пить. Ну никто не поверил, посмеялись надо мной.

**I** – Какой год то, 3 мая какого года?

**R** – Это был, так, то лет то прошло, наверное 5, больше. А потом значит, тот год не попил.

**I** – И они вам поверили. То есть, это 1-ый раз вы решили пообещать, то есть вы до этого никогда и не стремились бросать, да.

**R** – Ну, я СА, ну 3 месяца взял как-то ради эксперимента. Курить вот я, допустим, раньше очень легко бросал. Так это потом, значит, что там семейные передряги были, потом жена болела, отец умер, мать потом. Короче, и это, когда начинал курить, допустим, когда у жены выкидыш получился. Потом значит, отец умер, я закурил. Самое большое было 2 года я не курил, потом начинал. И с каждым разом все труднее бросить. Очень заразная привычка, а потом значит, мать меня закодировала. Она чувствовала, что умрет и говорит просто так пообещай мне, что если я умру, ты не закуришь. Я говорю: «Да живи ты, ради Бога». Ты что говорю, я все равно не закурю. Может быть ее поддержка какая-то там, не знаю. Потом мать умерла, мать похоронил. Так и это, ну просто все удивились, что я не закурил.

**I.** – И вам даже нравилось, вы гордились еще этим.

**R** – Да.

**I.** – А, вот когда пить то бросали, вы всем пообещали. Они вам не поверили, да.

**R** – Нет, сначала не поверили, а потом это.

**I** – Ну, вы вот как-то доказать им что-то хотели или для себя делали.

**R** – Ну нет, просто для себя решил, думаю смогу, не смогу, ради интереса, а потом, значит это, когда 3 мая, когда приближалось, жена ну, что это 3 мая пьянку закатим, год ведь будет у же как не пил. Я говорю - ладно, 3 мая не будем, 9 мая день Победы отметим. А у меня 28 мая день рождения, давай тогда 28 мая уж.

**Excerpt 1: English**

R: I had a bad hangover and decided to quit, I promised to my family

I: So your family asked you to quit?

R: No I didn’t really have long zapois, its like I get drunk once in a while, but then we had these 3 birthday parties in a row at work and then I decided from 3^rd^ May I would stop drinking completely, and no one believed me and people laughed at me

I: What year was it? That way 3^rd^ May of what year?

R: I am not sure but it could have been 5 years ago , maybe more. So from 3^rd^ May of that year, I didn’t drink till the end of the year

I: and they believed you? So this was the first time and before that you never had a desire to quit?

R: I decided to experiment, to not drink for 3 months, I used to quit smoking quite easily in the past, but then I had lots of family problems, first my wife got sick, then my father died, then my mother died, and so I just started smoking , for example when my wife had a miscarriage, then my father died, I started smoking again, the maximum time I stayed without cigs was 2 years, but then I started again, and my mother got me to do coding, she felt that she would die soon so she made me promise that if she dies I would not start smoking again, I said” By all means, you must live”. Don’t even talk about these things, I said to her, I am not going to smoke again, maybe that was her support, I don’t know, but then she died and we buried her but after that everyone was surprised that I didn’t start smoking again.

I: So did you like it, and were you proud of it?

R: yes

I: but when you stopped drinking, you promised in front of everyone and no one believed you ,

R: at first no one believed me but then

I: so you wanted to prove it to others for yourself

R: No I decided to do it for myself, i decided to test myself to see if I can do it or not, so when the 3^rd^ May was coming up she would hint that we have to dink on 9 May and on 28^th^ May I have my birthday and so let’s stop dinking after 28 May.

**Interview #12: Fluctuations**

**Excerpt 1: Russian**

R – Почти 20 лет я капли в рот не взял.

I – Да? Это с чем-то связано?

R – Связано, с сыном, старший, 80-го года рождения. Она собирается в школу, вот он в первый класс сейчас пойдет, а я выпивал тогда, че там завод

I – Это где-то 90-е года, да? Вы работали на заводе?

R – Это 87-й год, я на механическом заводе работал. И парнишка в школу собирается и еще мне мама сказала так. Я в садик пришел, ребенка нету уже, мама забрала моя, мы в одном доме жили, прибегаю домой, ребенок дома, все, сел и на столик голову положил. И вот ребенок этот подходит маленький, он раньше плохо говорил, папа пьяный, да, папа пьяный? Вот меня это заело. Невозможно было в то время толком вылечиться, в ЛТП, еще куда-нибудь. Я поехал в Москву в НИЦ, научно-исследовательский центр и там вшился, спираль, французская спираль тогда была. Мама у меня всю жизнь в медицине проработала и она перед этим врачом, вот я всю жизнь работала, ничего не получала и не просила никогда, вот ребенка моего спасите и вот мне вшили спираль. И я почти 20 лет не пил.

**Excerpt 1: English**

R: For almost 20 years I didn’t drink

I: Yes? What was that connected to?

R: It was related to my elder son, he was born in 1980. He is attending school, he will go for his first year at school, I was drinking then, factory doesn’t mean much to me

I: This was in the 1990s, yes? You worked in the factory?

R: This was in[1980s], I was working at the factory. The boy attends school, so my mother told me. So I went to the kindergarten but the boy was gone already, his mother had picked him up, well all lived in the same house, I ran home, the child was at home, I went and sat down at the table and lay my head down, and then the child came up to me, he was small and didn’t speak very clearly, and he asked me, you are drunk, you are drunk? I felt affected by this. It was impossible to get professional help to quit drinking back then. I went to Moscow to NITs, the scientific research centre and had a French *spiral* [unit placed inside body which releases toxins if you drink put inside of him] , my mother worked as a medic all her life, so she begging a doctor for me, that I really need it doing, then they put this spiral inside me and for almost 20 years I didn’t drink

**Excerpt 2: Russian**

R: yes, I started in the factory. I worked there before I was drafted to the army, then I got dismissed from the army and came back to the same place, I studied how to fix the remotely operated machines, basic computer programming, I was really happy about the job but then it became quite stressful, or maybe it just ran in the family because my father was always drinking, he was alcoholic all his life, he would say” I am a drinker, and always will be”, we buried him in 2000

I: and how old was he?

R: he was 71

I: so he was drinking strong stuff like vodka

R: yes up until his death

I: what about eau du cologne?

R: well as people would say, whatever was flammable, he would drink it , maybe that was influenced me, maybe not, all the time it was only housework, and it kept going on for years, only later we started to go on holidays with my wife, we went to Moldova to visit her relatives, so I noticed that I was drinking more and more but then I stopped

I: so you felt like you started to drink more, you had hangovers, or how else can you explain this process?

R: I had no limits

I: no limits? So you drank strong stuff

R: every time I would fix a piece of equipment they would give me a glass of vodka, 3 pieces of equipment, they would give me 25ml, then next 5 pieces 40ml

I: was that giddralizni spirit?

R: yes

I: that is technical spirit?

R: I don’t know, we were drinking it with sweet tea and it tasted good, everybody was drinking chemicals or glue, they would just drink basically anything

I: and how about yourself?

R: and I went down that way, I was working as a porter, I was working as a porter

I: were you drinking alone or with someone else?

R: well how can I put it I was drinking with all the lowlifes of the factory, this is how I hit rock bottom,

**Excerpt 2: English**

R – Я пошел на завод. И вот там я отработал, до армии отработал, в армию ушел, с армии пришел, опять сюда устроился. Выучился на наладчика станков с ЧПУ, 1-я группа выпускалась у нас на заводе по этому профилю. Нормальная работа, ну, че-то, не знаю, опять-таки нервотрепка, скорее всего, может быть, гены это, у меня отец по жизни алкоголик, говорил я пил, пью и буду пить. Похоронили мы его в 2000 году.

I – А сколько ему было лет?

R – 71 год

I – Ну, а пил он только крепкие напитки, водку там,

R – Все, в то время даже

I – одеколон?

R – Да все было, все, что горит, как говорится, пил. Может это повлияло, может что, все время дом – работа, дом – работа, месяцами, годами, в отпуск это мы уж потом стали ездить, с женой как-то пару раз съездили в Молдавию к родственникам, у жены там родственники были. Вот там что-то начал спиваться и в конце концов эту пьянку бросил (на заводе имеется ввиду).

I – А начали спиваться как, то есть употребляли много, или у вас было похмелье, или у вас, как Вы почувствовали, что начали спиваться?

R – нормы нет

I – нормы не было? То есть пили Вы тоже крепкие напитки в основном?

R – У меня на каждый станок – 8 станков – на 3 станка по 25 граммов дают, а на другие 5 станков по 40 граммов дают

I – Это гидролизный спирт? То есть Вы пили гидролизный спирт, да?

R – Да.

I – Он ведь считается техническим?

R – не знаю, с горячим крепким чаем сладким очень вкусно. Тогда все пили, политуру, клей, что только не пили.

I – А вы?

R – Да, потом дошел вот до этого, работаю стропальщик, грузчик, ну, то же самое, что грузчик.

I – А с кем Вы пили, один или с кем-то еще?

R – Это скажем так – дно завода, где перекидывают металл, вот до туда я скатился.

**Interview #13: Decrease**

**Excerpt 1: Russian**

R – В 2000 году я бросил пить совершенно. Я был страшный алкоголик, в 2000 году я бросил пить. И с этого момента у меня все пошло в гору, все, все, все, все, все. 2 года назад я бросил курить. Проверили у меня и кровь и печень, и кардиограмму снимали и сейчас у меня все хорошо. Я активно занимаюсь горными лыжами. Летом я очень сильно занимаюсь рыбалкой. Времени, естественно, свободного нет, юизнес, но такой активный отдых. В принципе чувствую себя очень, очень неплохо.

I – Если я Вас правильно поняла, то интервьюеры приходили к Вам, когда Вы уже бросили пить, да?

R – Да

I – они приходили к Вам не 2000 году, а гораздо позже?

R –Мгы

I – А на тот момент, у Вас уже была такая убежденность, что пить нельзя, если я правильно Вас поняла

R – Вы понимаете, параллельно со мной, мне 47 лет сейчас, мои друзья, с кем я шел параллельно, они не остановились и их нет уже, понимаете?

I – То есть друзья, которые были с Вами раньше, да?

R – Да

I – Те, которые были с Вами до 2000 года? И которые с Вами оставались Вы-то бросили пить, а они?

R – нет

I – Они не бросили вообще пить?

R – пробовали, пробовали, но у них не получилось

I – А Вы с ними продолжали общаться, так я поняла

R – Меньше общения было

I – У Вас было меньше общения

R – С ними стало меньше общения. С теми, с кем я встречался, можно сказать, что общения уже практически нет. Из старых друзей остались только самые самые

**Excerpt 1: English**

R: in 2000 I quit drinking altogether. I was a proper alcoholic and I stopped in 2000, from that point onwards my life was getting and better, two years ago I also quit smoking, they checked my liver, blood and heart and everything is fine now, I do downhill skiing, in the summer I fish a lot. I don’t have much free time, I have my own business, in general I am feeling very very good.

I: If I understand you correctly, the interviewers came after you quit drinking?

R: yes

I: and they came later than 2000

R: yes

I: and then you were already convinced that you should not drink, right?

R: You understand that I’m 47 now and my childhood friends, they didn’t stop, and they’ve gone already ( dead)

I: that s your childhood past friends

R: yes

I: Those you had before 2000? And did they actually stop drinking as well

R: no they didn’t

I: They didn’t stop at all?

R: They tried, tried hard, but did not succeed

I: but you still kept in touch with them, correct?

R: not as much as before

I: so who did you socialise with?

R: from socialising point of view, I lost of my friends from my old drinking days, from those days I only have hard core friends left

**Interview #14: Decrease**

**Excerpt 1: Russian**

R: Водку почти исключаю, пиво употребляю, меньше почти в 2 раза, только пиво стараюсь пить. Ребята смотрят. Младший говорит: «Папа ты, что купил? А пиво…», а если водку: «Смотри у меня».

I: Младшему сколько сейчас?

R: 7.

I: А старшему?

R: 12. Ну старший он как- то…, а младший смотрит за этим, младший больше смотрит за нами, как-бы обращает внимание. И хочется и колется, стараешься из двух зол выбирать и нельзя, не знаю как это объяснить. Работа есть, ребенок смотрит и отдохнуть хочется. Раньше я мог конечно выпить и на работе мог чуть – чуть, а сейчас…

I: А с чем Вы связываете, почему на работе не можете выпить?

R: Сейчас на работе всея ответственность на мне.

I: У Вас статус вырос, Вы стали мастером или прорабом?

R: Нет, я так и остался рабочим. Но просто ответственность, там все денежный эквивалент – надо отработать такую – то сумму. Мне начальник говорит: «Ты не справляешься».

**Excerpt 1: English**

R: almost no vodka for me now, only beer, and that is only half of my usual intake, so I ‘m trying to drink only beer, even my sons tell me off if I buy vodka from the shop, they check up on me

I: how old is your youngest son

R: 7

I: and the eldest son

R: he is 12

I: The elder son has a different reaction to what’s around him, the younger son pays attention to these things but the elder one notices much more, so I feel slightly guilty because if I want to drink in front of them it’s not easy any more, so you still have to relax after work, but you have your child watching over you, so you can’t really relax as you want to. Before I could drink a bit at work but now not really

R: so why do you think you can’t drink at work anymore?

I: Because I am much more responsible now

R: so you moved up the ladder, did you become a general manager, division manager?

I: no I stayed as ordinary worker, as before but these new responsibilities this is connected to my bonus so I get more money for extra work, so when the boss tells me you cannot really do it,

**Interview #15: Decrease**

**Excerpt 1: Russian**

I: Вы говорите пить меньше стали, а в связи с чем?

R: Смеется. Возможности меньше стало.

I: А что это за возможности?

R: Мало народу стало, с которым можно было почаще выпивать.

I: А куда народ делся?

R: Работают, кто не работает, кого нет вообще.

I: То есть компании у Вас не стало?

R: Распалась компания. А все остальное …

I: Насколько стали меньше пить? Раньше употребляли как часто?

R: Я не сравнивал, столько да столько, нет. Надо было, есть возможность- пил, не хотелось – не пил. Не то чтобы заставляли, будешь пить – буду, не будешь – не буду. Честно говоря.

I: Я так Вас поняла, что кого-то нет, с кем-то меньше видитесь, поэтому меньше пьете?

R: Да, сейчас и работы не стало, народу не стало, меньше так…

I: А раньше Вы на работе больше выпивали?

R: Да нет. Смеется. Ну можно было пойти на работу вначале смены пива выпил, в конце смены, пошли с работы - тоже выпил. А так больше нет.

**Excerpt 1: English**

I: you say that you reduced your drinking, but what is it connected to?

R: I don’t get so much opportunity any more

I: Opportunity for what?

R: there are very few drinking buddies that I can meet anymore

I: so where are the rest of them?

R: some of them are busy at work, some of them not working but still busy, and some of them are gone

I: so your circle of friends disappeared?

R: everybody drifted away , as for the rest

I: so exactly how did you reduce your alcohol intake? How much did you drink before?

R: I didn’t make any statistics on that, before if I had the chance I would drink. If I didn’t feel like I wouldn’t, nobody was forcing me to do it, this is my true word

I: from what I understand some people are gone so you don’t get to see them so often so this why you reduced your drinking

R: the situation with work is not that great, people are drifting away, so there are less chance to do it

I: so you were drinking at work before?

R: not really ( starts laughing) maybe at the beginning of the shift I would drink a little beer, maybe at the end a bit as well, but not really much

**Interview #16: Decrease**

**Excerpt 1: Russian**

I – Ага, от случая к случаю, по праздникам. Нормальное отношение у Вас к алкоголю было.

R – Сейчас крепких напитков стало поменьше, вот, стал пить где-то ну раз 6 за год, пиво, как бы слабоалкогольные напитки тоже поменьше. Ну, в целом как бы уже меньше.

I – То есть, все напитки стали пить меньше, как крепкие напитки, так и слабоалкогольные, как Вы назвали пиво, вино Вы не пьете?

R – Вино как бы нет. Я водку и пиво. Сейчас вот поменьше.

I – А..

R – Поменялся коллектив, все как-то задумались

I – Коллектив поменялся, Вы про работу, да?

R – Да.

I – А коллектив у Вас в основном, наверное, мужчины?

R – Ну, в основном да.

I – Вы сказали, что коллектив поменялся и что-то поменялось, а что?

R – Сейчас все стали как бы более ответственно, медики проверяют

I – А раньше они относились к этому неответственно?

R – Послабления были.

I - А в чем это выражалось?

R – На с как бы медик каждый день осматривает давление, ну, если ты пришел там с остаточным запахом, могли наругать,

I – Медик Вас проверял каждый день, а сейчас?

R – Также проверяет,

I – А в чем тогда послабление? Раньше были послабления?

R – раньше были, вобщем с запахом пришел, раньше могли поругать, то сейчас попался – выговор, еще раз попался – уволили.

I – Во-первых, это строго стало на работе, стали строже спрашивать..

R – стали ответственнее относится

**Excerpt 1: English**

R: not many strong drinks around anymore, if I drink, I drink maybe 6 times a year, mostly beer, but I’ve noticed that even low volume weak like beer I don’t drink so many

I: so you really slowed down on the spirits and on weaker drinks as well, like wine or beer? Do you drink these?

R: I don’t drink wine, I drink vodka and beer, but,much less now

I: Ah

R: My circle of friends at work changed and they all started to be concerned about it

I: you mean at work?

R: yes , your work mates are all men, is that right?

I: mostly yes

R: but you said your work colleagues changed, what exactly changed?

I: everybody got much more responsible now, and the medics sometimes check us

R: and before you were not so responsible for your health?

I: yes, you could say that

R: and how did it manifest itself?

I: there are some qualified medical staff checking our blood pressure, and if they smell alcohol on your breath you could get into trouble

R: that was in the past when they checked everyday, what about now

I: they still check us now

R: so why were you more relaxed about it in the past? How was it back then?

I: well my work was more relaxed, when I would come smelling of alcohol they would just slap your wrist but now it’s all official, first they give you a warning and if you get caught for the second time you lose your job

R: first it became tougher at your workplace, they got much stricter about it

I: yes it’s all much more responsible now

**Excerpt 2: Russian**

I – Понятно, а все-таки почему, как я поняла, Вы знали свою дозу, относились к этому нормально, а почему Вы меньше стали пить?

R – Опять же от коллектива многое зависит.

I – То есть Вы считаете, что в коллективе очень многие бросили и поэтому и Вы тоже стали…

R – Раньше бывало кто-нибудь подойдет, давай, по пивку. Ну, давай. 1 бутылочка, две, потом может и водка пойти. То сейчас как бы многие пить перестали или тоже стали меньше пить и поэтому таких предложений стало меньше поступать, вот исходя из этого.

I – А предлагали давай по пивку те же, с кем вы сейчас работаете?

R – Почти те же самые.

I – Ну, они не ушли из коллектива, работают, но уже не предлагают.

R – Кто ушли, кто-то сам не хочет сейчас.

I – А с тем, которые ушли, Вы с ними не поддерживаете отношения?

R – Как бы нет, но иногда по делам что-нибудь узнать. Основная масса как бы осталась, с кем я общался.

I – Большинство-то осталось, да?

R – Да, то, что коллектив поменялся, в основном как бы молодежь ушла, на место нее новую молодежь приняли, а с кем я общался, в основном они те же самые остались. 1 человек пришел. А так в основном половина зависит от коллектива.

**Excerpt 2: English**

R: It all depends on your work atmosphere

I: So you were influenced by people at work and started to drink less

R: yes in the good old days someone would come and offer a beer or two or and maybe some vodka too, but now all these guys they either stopped altogether or drinking much less than before, so it’s not likely you will be offered anything

I: the people who used to work with they would offer you a beer

R: always the same people

I: so the people stayed the same but they don’t offer a beer any more

R: well some of them are gone and some them don’t feel like drinking themselves these days ,

**Interview #17: Decrease**

**Excerpt 1: Russian**

R: а вот это..алкоголь я могу бросить без проблем. Однажды было как-то даже так..мы поспорили. Моя жена с подружкой ..я муж у подружки. Они, две подружки задают нам вопрос а слабо вам бросить пить. – Никаких проблем! Я два года не пил, не хотел даже...

I: Когда это было?

R: Это было примерно...так (задумывается)..в третьем году у меня брат умер. Вот с его похорон я начал пить снова...тогда и выпил первый раз, после похорое.2003 год был, значит. Значит, перестал в 2000-2001

I: А это как-то на спор было?

R: просто так, без всего – вам слабо? А мы с ним так, частенько встречались и это вот...выпивали. Вот он нам и задали вопрос – вам слабо бросить? Так нет! Никаких проблем

I: оба не пили7

R: Да. Он до сих пор не пьёт, ему понравилось. А я бы тоже не пил, но у меня проблема получилась с братом...Мал того, что мы братья, мы еще и с детства друзья. А там – немножко на похоронах, потом 9-й день, потом 40-й день, так и пошло уж. Потихонечку, потихонечку..сейчас снова начал как раньше. Ну в принципе ведь я не увлекюсь этим..

I: в неделю сколько раз бывает?

R: ну часто бывает раза три, но я пью немножкл

I: нуВы водку пьёте?

R: да, только я водочку..грамм 100-150, не больше..Вышел например вот сегодня (с работы), вот сейчас вот поехал, товарища вот заберу, заеду еще кое-куда с ним, поеду в огород, и там выпью грамм 150. Пусть даже я в огороде, хоть и идти больше никуда не надо, но мне больше не надо. Для настроении, чтобы можно было поговорить, для веселья

I: но это с компанией?Жена с Вами не выпивает

R: нее..она вообще не пьёт. Ей нравилось, когда я совсем не пил.

I: Она не особо активно борется с тем, что Вы выпиваете?

R: я ведь этим самым не мешаю ей. Она вог даже сама говорит «Юра, если выпивает, он ведь в меру пьёт». Вот выпью и всё. Вот будет стоять, а я даже не буду пить. Не надо мне

**Excerpt 1: English**

R: But alcohol I can quit no problem, We even had a wager on this one, my wife’s girlfriend and her husband came over and they dared us that we couldn’t possibly quit drinking, and then we stood up the challenge, and for 2 years I didn’t touch alcohol.

I: When did that happen?

R: That was approximately ....in 2003, my brother died, so form the moment of his funeral, I started drinking again, and then I drunk on his funeral, that was 2003, meaning that I stopped in 2001,

I: so was it for a lot of money ?

R: no just for fun, they just dared us to do it, and before we used to get together for a drink, they posed this question, and it wasn’t a big question

I: so you both stopped drinking

R: this other guy, he still doesn’t drink. He really liked it. I would have continued being sober if it wasn’t for the death of my brother, he hasn’t just my brother, he was my best mate since childhood, then we had the funeral service after 9 days, after 40 days, then just drinking slowly but surely since then. So I went back to the 2001 level but I shouldn’t have

I: and how many times a week do you drink?

R: quite often its 3 times a week but the dosage is quite small

I: are you drinking vodka?

R: yes, only vodka, between 100-250 ml, no more than that. For example, I have drunk today , I am going to pick up my mate now, and go somewhere for a bit of a drink, and then I’m going to my allotment, and maybe I’ll have another 150ml there, it’s just to improve my mood, its just that its easier to talk with people and joke

I: but this is in company. What about your wife? Does she drink with you?

R: No, she stopped completely, and she really liked it when I was sober

I: So she wasn’t really against your habit?

R: it doesn’t really bother her, it doesn’t interfere with her. She sometimes says to me “Yuri, even if he is drinking, he doesn’t overdo it”, I know my dosage, even if it’s in frnt of me, if I’ve had enough, I won’t over do it

**Interview #18: No change /surrogate drinker**

**Excerpt 1: Russian**

R: не, в 94-м я работал, в [*factory*] работал. А в трест столовых я где-то в 86-97..где-то так вот я..там начал пить

I: а пили после работы, с друзьями?

R: после работы, в 6 часов кончаешь работу, машину ставишь, за закуской сходишь

I: и часто? И каждый день это было?

R: почти каждый

I: у Вас бывало такое в жизни, что Вы пили и Вы понимали, что слишком много пьёте?

R: с друзьями ак стоишь, вокруг машины-то, в картишки поиграешь, всё, там, расслабляешься, вот – в 12 часов – домой, на такси, утром на работу

I: то есть Вам никогда не казалось, что чересчур и надо поменьше пить?

R: дак мы это, там мало пили,

I: ну, а, допустим, сейчас, когда Вы пьёте, у Вас утром бывают какие-то неприятные ощущения?

R: ну, похмелья не бывает, нет. Ну, если когда смешаешь, ну там – водка, портвейн, пиво – вот тогда бывает. Ну, как ерша...вот тогда..голова, семь на восемь, бошка,

I: а вот Вы когда-нибудь пили эти вот..всякие..

R: фуфырики?

I: ну да Боярышник всякий..

R: фуфырики – пробовал. Но это неприятно. Ясон пил, Композицию, Боярышник,

I: а не помните, когда это было? В какое время?

R: Перцовку я недавно пил, где-то месяц назад, хорошая штука.

I: Вы покупаете, то есть?

R: зачем? Ребята..там сидят, говорят – будешь? Я – давай. Говорят – да давай – хорошая, хорошая.

I: а Вы разбавляете её или так пьёте?

R: разбавляем

**Excerpt 1: English**

R: yes in 1994 I was working in [*factory*] but when I started my work in 1986 in a restaurant this is when I started drinking

I: so you were drinking after work with you friends

R: after work we would finish at 6, park the car then get ourselves together to celebrate

I: how often was it on a daily basis

R: almost daily

I: so it came about it your life that you realised that you are drinking too much.

R: No we had a great time we were really socialising and relaxing together and we would go home by taxi so we have enough sleep for work the next day

I: so it didn;t occur to oyu you were drinking too much and oyu should reduce your intake

R: not really we didn’t drink taht much

I: but now how do you feel in the morning when you drink

R: if you mean, hangover, I don’t get them , it only happens if you mix drinks

I: but at some point have you ever drunk...

R: surrogates?

I: yes have you tried Boyarashnik

R: from the pharmacy I have tried Yason, Kompositsaya, Boyarashnik, but this is all horrible stuff

I: when exactly was it?

R: recently I tried Persovka, one month ago, that stuff was good

I: so you go and get it yourself

R: not really my work mates offer it to me

I: do you mix it with anything?

R: yes we mix it

**Interview #19: Decrease**

**Excerpt 1: Russian**

I – В семье у Вас и никто не пьет.

R – Нет, а у меня пацаны совсем не пьют. Они меня все время совестят

I - А, это они Вас совестят? Это хорошо или плохо, что они не пьют?

R – Да, Наверное, хорошо.

I – Совсем не пьют?

R – младший у меня барменом когда работает, он сам разливает, они в компании бурду не пьют, знают, что такое хорошее вино, ну, для удовольствия, как говорится. А старший вот практически, ну, может быть на день рождения, да че да.

I- И не курят?

R – курить-то оба курят, но мне не показывают.

I – Скрывают от Вас, да?

R – Да.

I- А так, они призывают Вас к тому, чтобы Вы не пили. И что говорят интересно?

R – Ну, что говорят, пьешь много говорят, че говорят.

I- Так и говорят, что много пьешь, так вы вроде немного пьете, 250 и не каждый день.

R – все равно считается…

I – Они считают, что много пьете?

R – Да

I – А вы считаете, что немного пьете? А вы с ними спорите, или соглашаетесь иногда?

R – А че спорить-то, отшучиваюсь.

I – Жена как?

R – Жена, она то же самое, че…

I – Тоже бывает, что совестит, да?

R – Да. Тоже говорит, что много

**Excerpt 1: English**

R: No, my boys don’t drink at all, they are always guilt tripping me because of that

I: Oh, so they make you feel guilty? Its good or bad, that they don’t drink?

R: yes for sure, its good

I: so they don’t drink at all?

R: when the youngest works as a barman he knows good stuff from bad stuff, and his circle of friends they wouldn’t drink any rubbish, so they all know what good wine tastes like, they drink it for pleasure, And the oldest practically doesn’t drink, maybe on birthdays or something

I: and they don’t smoke?

R: they both smoke, but they don’t let me see it

I: they hide it from you?

R: yes

I: and so they try and dissuade you from drinking, are you interested in what they say?

R: well they say that I drink too much and that’s it

I: well they say you drink too much, but you don’t drink that much, only 250ml from time to time

R: well they still think...

I: that’s it too much?

R: yes

I: and do you think it’s too much? And do you argue with them about it?

R: we argue, but I just joke about it, there is no point arguing

I: what about your wife?

R: the wife thinks the same..

I: so she also tries to nag you?

R: yes, she also say I drink too much

**Interview #20: Decrease**

**Excerpt 1: Russian**

I. – Ну, вобщем-то вас испугали тогда?

R. – В принципе испугать-то, че испугали? Я и сам решил бросить.

I. – Раньше-то частенько выпивали, да?

R. – на полетах-то? Мы нашли таблицу – степень алкоголизма. Там че хочешь найдешь же. Там вот 14 степеней алкоголизма. Самая маленькая там, по-моему, кружку пива в неделю, 1-й степени алкоголизм. Ну, вобщем, там той стадии нету, что мы пили

I. – зашкаливало, да

R. – зашкаливало, да. Вобщем посмеялись мы. На строе было. Тем более инспектора эти. Не керосинный двигатель. Вся очистка эта, вся на спирту.

I. – и заправка что ли на спирту?

R. – Ну, вобщем там баллон такой есть, час полетов – литр спирта списывается. Вот получают на полет 2 канистры по-моему по 20 кг, ну, 50 литров на полеты, вот считайте.

I. – он технический?

R. – нет, когда как. Иногда пшеничная водка попадает, там ведь тоже проверяют. Там же цистернами приходит, сразу проверяют. Если цистерна была из под мазута, спирт не берут, раз-два откажут. Это же в Советском Союзе, это же самолеты. Если что-то откажет, начинают искать. Короче, спирт чистейший приходит туда, технический другой спирт. Попадет технический – радиостанция работает не так, это же не машина и стекла запотеют, что-то если попадет техническое. Написано в ГОСТе, что положено.

I – А там контора-то следила за тем, выпивает или не выпивает личный состав?

R. Конечно, как без контроля?

I – И как полагается, закуска была при этом, чтобы без запаха?

R. Когда как. А так понедельник, среда, пятница – полеты. 1 раз – 3, 1 раз – 4 в неделю полеты. Перед полетами летчиков-то каждый раз проверяют, они проверку проходят перед полетами. Доктор уже знает, что если у него давление Вот унего 120/70 было, сегодня пришел 125/85 – все, что-то не то значит. Летуны пили только в пятницу. Вот в пятницу полет отлетывают, в субботу, воскресенье выходные, вот они эти два дня отлеживаются. Нам-то проще, нас то че, не проверяют.

I. – все равно большого пьянства наверное не было, надо все равно соображать.

R – ну, да

I. – Ну и что изменилось после того, как вы перестали, прекратили выпивать?

R. – все сменилось. Друзья-товарищи. Тот контингент там остался, тут другие все. Все сменилось, вообще все.

**Excerpt 1: English**

R: In principle I was scared, but scared of who? I decided to give up myself.

I: so before you drank more often?

R: on flights? We found a table on degrees of alcoholism. There you can find where each person fits. There are 14 degrees of alcoholism, it said there. The lowest degree is, in my opinion, drinking a glass of beer a week, this is the 1^st^ degree of alcoholism. Well, in general, there was no stage for what we were drinking.

I: so you drank pretty often then?

R: often, yes. In general we had a good time. That was how it was. Especially those inspectors, it was a non-kerosene engine, all that cleaning, they were all on spirits.

I: so were they filling up on alcohol?

R: Well, basically, there is a tank there, on an hours’ flight, a litre of alcohol is written off. So on each flight they get 2 canisters of 20g, so , 50 litres in a flight, if you consider

I: is it technical spirit?

R: no, it depends, sometimes wheat vodka, but they check for that. It comes in cisterns, they check it straight away. If the cisterns were used for fuel oil, they don’t take the alcohol, once or twice they refused. This was in the Soviet Union, these are planes. If somebody refuses, they start searching for reason. Basically, it was clean spirit there, technical is another kind. If technical spirit goes, the radio doesn’t work well, because it isn’t the machine and the glass gets steamed up if some technical will be added. It was written in GOST (state standard – LS), what you needed. .

I: so what did the company do about drinking or not drinking of the staff?

R: Of course , how can they leave it uncontrolled?

I: but as expected, there were some snacks with the drink, so that there was no smell of alcohol?

R: It depends. There were flights on Monday, Wednesday and Friday. One time, three, one time, 4 flights a week. Before flying the pilots were checked every time, they had to go through a check before flying the plane. The doctor already knows, that if they had a blood pressure of 120/70 and today its 125/85, that is doesn’t mean much. So pilots drank only on a Friday. So on a Friday he flies the plane, and on Saturday and Sunday it’s the weekend, so they have 2 days resting. We have something simpler, and they don’t check

I: so it doesn’t matter , there wasn’t more drunknenness in the past, but all the same you need to think about it

R: well, yes

I: were there any changes after that, when you gave up or cut down on your drinking?

R: Everything has changed. My friends and colleagues. There is a contingent who stayed the same, but all the others are different. Everything changed, basically everything.

**Interview #21: Decrease**

**Excerpt 1: Russian**

R: Родилась еще одна дочь, финансы наладились, работа стабилизировалась. На алкоголь времени совсем нет, да и пустое это занятие.

I: Вы стали считать это занятие пустым? После чего?

R: [date] 2010 года родилась младшая дочь, после ее рождения все поменялось.

I: Что поменялось?

R: Стрессов не замечаю, они есть конечно, но я смотрю на события по- другому.

I: Жизнь воспринимается более ровной и стабильной?

R: Я познал несколько истин: 1. Никому не завидовать, не гнаться ни за кем и не за чем. Я раньше гнался за деньгами. Многого не замечал. Деньги должны доставаться с потом, тогда они «вкусные».

**Excerpt 1: English**

R: I had another daughter born, my financial situation improved, things got better at work, I don’t have time for drink, and drinking is just a waste of time

I: you think this is just a waste of time? What happened to make you think like that?

R: on [date] 2010 I had my youngest daughter born and after that things changed,

I: what exactly has changed?

R: I don’t pay attention to small irritations in life and look at things differently

I: So you perceive life as a much more stable thin

**Interview #22: Fluctuations**

**Excerpt 1: Russian**

I: Как по вашим ощущениям, лет пять назад потребление алкоголя у вас было меньше, больше?

R: Тогда меньше было. С 2003 по 2008 я вообще не употреблял. Я кодировался.

I: А на кодировании кто настаивал, жена?

R: Жена, давай, говорит, сходи. Я не против был. На Молодежной жил, там друзей было полно, приходили (выпить), канистрами (пиво) покупали.

I: То есть вы квартиру поменяли?

R: Да, но это в 2002 или 2003 было. Кажется в феврале 2002 мы заехали, зима была.

I: Значит переехали и решили начать новую жизнь?

R: Да, да. Тут один человек, пенсионер, говорил, что ему квартплату тяжело платить, давай поменяемся. Поменялись. Свою двухкомнатную отдали ему, а эту [*his employer*] выделила с доплатой в 400 тыс. (четырехкомнатную)

I: То есть вам на работе ее дали как раньше, только с небольшой доплатой как с неполной стоимостью.

R: Да, как беспроцентную ссуду.

I: И как с кодированием?

R: Нормально. 5 лет не пил. Срывов не было. в 2003 [dates], закодировался на[*central location in Izhevsk*] , в подвале у них там. Там многие приходили кодироваться: женщины, молодые девушки. Потом в 2008 попробовал бутылочку пива, но мне плохо стало, тошнило, рвало.

I: С тех пор получается, что сильной тяги у вас нет, в руках себя держите?

R: Да, могу немного выпить.

I: Как думаете, это лечение помогло или какая-то другая причина.

R: Мне, по-моему, лечение. Кто кодировался и срывался, то начинали пить много, а у меня такого нет.

**Excerpt 1: English**

I: how would you evaluate it, did you drink more or less than 5 years ago?

R: It was less. Between 2003 and 2008 I didn’t drink. I did coding.

I: and who insisted you had coding, your wife?

R: My wife said go and do it. I was against it. When I lived in Molodeshnii I would get together with friends, come and drink, bring cans of beer

I: have you changed your apartment?

R: Yes, but that was in 2002 or 2003. I think it was 2002 we moved, in winter.

I: so you moved and decided to start a new life?

R: yes, yes. Then one man, a pensioner said it was hard to pay his rent, so lets switch. We switched. We have him my own 2 room apartment, and [*his employer*] subsidise it by 400 thousand ( 4 room apartment)

I: So as before work give you your salary, only with slightly less each month?

R: yes, its like an interest free loan.

I: and so what about the coding?

R: OK. For 5 years I didn’t drink. It wasn’t any disruption. In 2003 [dates] I was coded in [*central location in Izhevsk*]. Many people go there for coding: women, young women. Then in 2008 I tried a bottle of beer, but I was got very bad, sick, vomiting

I: And since then how has it gone, you didn’t have a strong urge, you’ve got it all in hand?

R: yes, I can drink a little

I: what do you think, that the treatment helped or it was some other reason.

R: I think it was the treatment. There are those who get coded and break it, and start to drink a lot, but I didn’t like this.

**Interview #23: Decrease**

**Excerpt 1: Russian**

R: да пиво, водку..вина – не..не знаю, я задыхаюсь с вина, что ли..Одно время было как-то там..в пеестроечные времена..было время – всякую ерунду пили..всякие там портвейны, я задыхался почему-то..всё равно всё эо не натуральное, 72-й портвейн, да всё..всё красители всякие,

I: вот Вы говорите, что последние лет десять ничего не менялось с алкоголем. А вот когда Вы без работы были, было ли такое, что больше выпивали?

R: не, вот когда перестройка началась, в эти 90-е года, вот это да – было.Вообще завод ведь не работал. Неделю можешь работать, полмесяца не работать. А то и месяц не работал. Силишь дома, тебе звонят – вот – немножко, немножко вот это..типа индивидуалы такие..во надо – за столько-то зарплаты надо машину укомплектовать Пришёл, сделал, ушёл.Опять сидишь на телефоне.

I: и тогда больше выпивали, да?

R: кивает

I: а в последнее десятилетие – нет?

R: дак..у меня вот даже бывший друг, бригадир, даже повесился от такой жизни.

I: от какой?

R: ну вот, от перестройки.

I: он как, по пьянке повесился?

R: ну, он, трудоголик был, конечно. Он выкладывался на работе.

**Excerpt 1: English**

R: we drink beer and vodka, I have strange side effects from wine... I get very short of breath, one occasion, that was before, at the time of perestroika, then we were drinking all this rubbish like port and I was feeling very short of breath because its full of chemicals, portwine no.72, it has artificial colouring

I: so in the last 10 years nothing has changed in your drinking, so when you were unemployed did you tend to drink more?

R: when perestroika started and in the 1990s yes it happened. In general the factory was not working very well. Work was very patchy, we could work for one week then not again for the next fortnight. Sometimes we didn’t work for the whole month. So you would stay at home and people would call me, they would say just a little, such selfish people, and they would say to you, for this amount of money, can you go and fix the car? So you would come, fix it, and leave, and then you would just sit in the house and wait for the next call

I: and then you were drinking more?

R: yes

I: and in the last 10 years it changed?

R: yes, I had a friend who killed himself because of this lifestyle

I: what kind of lifestyle?

R: he killed himself because of perestroika

I: so he got drunk and killed himself?

R: oh he was a proper workaholic, he would put his heart and soul into his work

**Interview #24: Fluctuations**

**Excerpt 1: Russian**

I: ну вот год Вы не пили..А поом что произошло? Почему год? Почему не продолжили?

R: у меня машина была..перевернулся..и после эого вот..

I: машина была?

R: да

I: а говорите – никаких событий не происходило..когда это было?

R: это было давненько..

I: можете вспомнить? Год, когда авария-то – трудно забыть..

R: ну, лет 10 назад..Я вот тогда год не пил..и вот тогда не пило..и сейчас тоже самое

I: то есть, Вы уже не первый раз кодировались?

R: да, это уже не первый раз..

I: тогда давайте вернёмся к тому году..Вы тогда закодировались., не пили а потом машина перевернулась.

R: Ну да..мать умерла в больнице

I: а Вы вместе с матерью были?

R: ну да..мы всей семьёй были..

**Excerpt 1: English**

I: so the whole year you didn’t drink, why just one year, what happened?

R: I had a car and I had an accident, and after that I...

I: so you had a car accident?

R: yes

I: and you said that nothing happened...When did this happen?

R: that was quite a while ago

I: can you remember things like accidents are very easy to forget

R: about 10 years ago , before the accident I didn’t drink for a whole year and it’s the same now

I: so it’s not the first time you had coding then ?

R: no

I: well lets go back to the events of that year when you had coding done , then you didn’t drink and then you had this car accident

R: yes and my mother died in hospital

I: so you were together with your mother in the accident?

R: yes the whole family was together in the accident

**Interview #25: Decrease**

**Excerpt 1: Russian**

R. Я в меру употреблял, но выпить любил

I. Бутылку водки могли выпить?

R. Ну, бутылку-то, наверное, нет, по 259 граммов выпивал все время

I. Запоев не было?

R. Запоев нет, не было

I. Сейчас меньше стали пить

R. Сейчас вообще не пью, с 28 числа

I. Страх появился что ли

R. Ну, страх не появился, а зачем, хотя шампанское оставили мне бутылочку. У меня коньяка было много, специально купил и шампанское. Думаю как, встретим Новый год и по гостям поедем со своим коньячком и шампанским, Ну, так стоит шампанское, но не рискнул пить его, не рискнул. Ну, страха нет, просто не хочу, тем более таблеток столько пью

**Excerpt 1: English**

R: I drank to some degree, but I wasn’t a heavy drinker

I: Could you drink a bottle of vodka?

R: well, probably not a bottle, but 250 gms I drank usually

I: did you have zapois?

R: zapois never

I: now you drink less

R: now I don’t really drink, from the 28^th^

I: because you got scared?

R: no, I wasn’t scared, why, I still have a bottle of champagne standing there. I had a lot of cognac bought specially and champagne. I think that we’ll get to New Year and guests will come and bring their own cognac and champagne. So, the champagne stands there, I won’t risk drinking it. Well, it’s not fear, I just don’t want to, I take so many tablets now
